# Peripheral blood mononuclear cell respiratory function is associated with progressive glaucomatous vision loss

**DOI:** 10.1038/s41591-024-03068-6

**Published:** 2024-06-17

**Authors:** Bledi Petriti, Alessandro Rabiolo, Kai-Yin Chau, Pete A. Williams, Giovanni Montesano, Gerassimos Lascaratos, David F. Garway-Heath

**Affiliations:** 1 NIHR Biomedical Research Centre, Moorfields Eye Hospital and UCL Institute of Ophthalmology, London, UK; 2 Department of Clinical and Movement Neurosciences, UCL Queens Square Institute of Neurology, London, UK; 3 Department of Health Sciences, Amedeo Avogadro University of Eastern Piedmont, Novara, Italy; 4 Department of Clinical Neuroscience, Division of Eye and Vision, St. Erik Eye Hospital, Karolinska Institutet, Stockholm, Sweden; 5 King’s College Hospital NHS Foundation Trust, King’s College London, London, UK

**Keywords:** Prognostic markers, Neurodegeneration

## Abstract

Intraocular pressure (IOP) is currently the only modifiable risk factor for glaucoma and all licensed treatments lower IOP. However, many patients continue to lose vision despite IOP-lowering treatment. Identifying biomarkers for progressive vision loss would have considerable clinical utility. We demonstrate that lower peripheral blood mononuclear cell (PBMC) oxygen consumption rate (OCR) is strongly associated with faster visual field (VF) progression in patients treated by lowering IOP (*P* < 0.001, 229 eyes of 139 participants), explaining 13% of variance in the rate of progression. In a separate reference cohort of untreated patients with glaucoma (213 eyes of 213 participants), IOP explained 16% of VF progression variance. OCR is lower in patients with glaucoma (*n* = 168) than in controls (*n* = 50; *P* < 0.001) and is lower in patients with low baseline IOP (*n* = 99) than those with high baseline IOP (*n* = 69; *P* < 0.01). PBMC nicotinamide adenine dinucleotide (NAD) levels are lower in patients with glaucoma (*n* = 29) compared to controls (*n* = 25; *P* < 0.001) and strongly associated with OCR (*P* < 0.001). Our results support PBMC OCR and NAD levels as new biomarkers for progressive glaucoma.

## Abstract (web summary)

Reduced mitochondrial respiration in peripheral blood cells is associated with faster vision loss in glaucoma patients with controlled intraocular pressure.

## Main

Affecting ~80 million people by the end of this decade, glaucoma is a chronic, progressive optic neuropathy in which retinal ganglion cells (RGCs) die, leading to vision loss. It is the leading cause of irreversible blindness worldwide^1^. High intraocular pressure (IOP) and older age have been established as the most important risk factors for glaucoma and its progression, with IOP being the only modifiable risk factor^2,3^. While lowering IOP slows the rate of visual field (VF) progression^3–5^, up to 60% of glaucoma patients with European ancestry^6^ and up to 90% with Asian ancestry^7^ lose vision despite IOP being within the normal range (normal-tension glaucoma (NTG)). Many patients still progress despite IOP lowering or meeting target IOP^8,9^ with 38.1% blind in one eye and 13.5% blind in both eyes 20 years from the initial diagnosis^10^. This suggests that other factors confer susceptibility to glaucomatous neurodegeneration^11^ and underlines the importance of identifying new, potentially treatable, risk factors. Several studies have indicated that altered mitochondrial bioenergetics is associated with both the presence, and resistance to the development, of glaucoma^12–22^. However, to date, no studies have shown the extent to which mitochondrial function is associated with glaucoma progression and whether it can be used as a biomarker for predicting faster disease progression. In trying to understand potential causes and consequences of mitochondrial dysfunction for neurodegeneration, several research groups have investigated nicotinamide adenine dinucleotide (NAD)—an essential cofactor central to mitochondrial function, ATP synthesis, cellular metabolism and a key regulator of axonal health^23,24^. NAD levels decline in the retina of glaucomatous DBA/2J mice in an age- and IOP-dependent manner, rendering RGCs susceptible to IOP-related stress and accelerating glaucomatous neurodegeneration^15^. Patients with glaucoma have been reported to have low levels of nicotinamide (NAM; the amide form of vitamin B_3_, an NAD precursor via the NAD-salvage pathway) in sera^25^. Furthermore, population-based studies have found that lower niacin (vitamin B_3_) intake was associated with NTG^26^, and greater niacin intake may be associated with a lower chance of developing glaucoma^27,28^. The role of NAD in mitochondrial respiratory function suggests that bolstering cellular NAD^+^ levels may improve cellular energetics and stress responses in neurons. The potential for modifiability of NAD levels is evidenced by the results of preclinical glaucoma models^15,29^, and short-term clinical trials show electrophysiological and visual function evidence of recovery of RGC function with high-dose NAM supplementation^18,30^. Several clinical trials have now been initiated to evaluate the potential of high-dose NAM to slow VF loss in glaucoma (for example, the nicotinamide in glaucoma (NAMinG) trial—EME NIHR132758, NCT05405868, and the glaucoma nicotinamide trial (TGNT), NCT05275738).

Mitochondrial function and NAD levels may decline as a result of generalized changes, such as aging, or may be a consequence of local IOP-related stress in the optic nerve head (ONH). As the tissues of the ONH are not accessible in vivo, we assessed the generalized aspect, using peripheral blood mononuclear cells (PBMCs) as the model system for bioenergetic analyses.

We provide evidence that PBMC mitochondrial respiratory function is lower in primary open-angle glaucoma (POAG) than in age-similar nonglaucomatous controls and is even lower in NTG compared to high-pressure POAG (high-tension glaucoma (HTG)). Furthermore, we demonstrate a strong association between PBMC mitochondrial function and the rate of VF progression. We also demonstrate that total NAD levels are lower in POAG PBMC and that NAD levels correlate positively with mitochondrial respiratory function providing new biomarkers for progressive glaucoma.

## Results

### Patients with glaucoma have reduced mitochondrial respiration

**Fig. 1:**
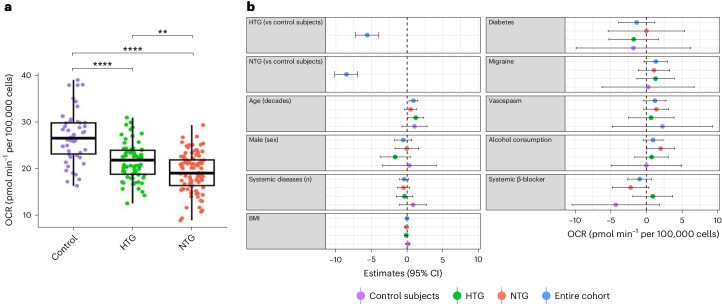
Mitochondrial respiration in PBMCs. **a**, Box plots and Tukey HSD post hoc test for basal OCR in PBMCs as a function of diagnostic category. OCR was measured using the XFe24 Analyzer in 218 participants (50 controls, 69 HTG and 99 NTG). NTG patients had the lowest basal OCR (mean (s.d.): 19.2 (±4.1) pmol min^−1^ per 100,000 cells) followed by HTG (mean (s.d.): 21.6 (±3.9) pmol min^−1^ per 100,000 cells) and controls (mean (s.d.): 26.8 (±5.5) pmol min^−1^ per 100,000 cells). The box plots display the distribution of basal OCR values within each diagnostic category. Each box plot represents the IQR of the data, with the horizontal line inside the box indicating the median value. The lower and upper bounds of the box represent the first and third quartiles, respectively. The ‘whiskers’ extend to the minimum and maximum values within 1.5 times the IQR from the lower and upper quartiles, respectively. One-way ANOVA with Tukey’s HSD test to control family type I errors for post hoc pairwise comparisons. No adjustment for multiple comparisons was made for all other tests. Specifically, NTG versus control comparison yielded a *P* value of <0.0001, HTG versus control comparison yielded a *P* value of <0.0001 and HTG versus NTG comparison yielded a *P* value of 0.001. Significant differences with **P < 0.01 and ****P < 0.0001. **b**, Forest plot shows the results of the multivariable linear regression model for factors associated with basal OCR in 218 participants (50 controls, 69 HTG and 99 NTG). Blue, red, green and purple circles represent β estimates from the multivariable regression model, and the horizontal bars represent the corresponding 95% CIs. The model predicts that having NTG is associated with basal OCR being 8.5 pmol min^−1^ per 100,000 cells lower than controls (*P* < 0.001) and having HTG is associated with basal OCR being 5.6 pmol min^−1^ per 100,000 cells lower than controls (*P* < 0.001).

Mitochondrial respiration was measured as oxygen consumption rate (OCR) using the XFe24 Analyzer in PBMC of 218 participants (99 NTG, 69 HTG, 50 age-similar controls; Table 1; participant details). NTG patients had the lowest basal OCR (mean (s.d.) pmol min^−1^ per 100,000 cells): 19.2 (4.1), followed by HTG 21.6 (3.9) and controls 26.8 (5.5; Fig. 1a). The differences HTG and NTG versus control (*P* < 0.001) and NTG versus HTG (*P* < 0.01) were all statistically significant. Maximal OCR, ATP-linked OCR and reserve capacity followed the same trend (Supplementary Fig. 1). The intra-assay coefficient of variation (CV%) between technical repeats for basal OCR was 10%. Repeatability was measured in 31 NTG and 12 HTG participants on two occasions. The median (interquartile range (IQR)) number of days between test and retest was 217 (126–746). The mean (s.d.) basal OCR was 20.8 (6.6) pmol min^−1^ per 100,000 cells. Here 95% of values were within −6.5 and +6.2 pmol min^−1^ per 100,000 cells of the test–retest mean difference (−0.1 pmol min^−1^ per 100,000 cells; Supplementary Fig. 2).

**Table 1:** Demographic and clinical characteristics of patients with NTG, HTG and healthy controls

Variable	NTG	HTG	Control	*P* value
Number of patients	99	69	50	
Age, mean (±s.d.), years	72.3 (± 10.1)	73.6 (± 9.6)	68.4 (± 10.1)	**0.018**
Sex, male/female, *n*	63/36	37/32	29/21	0.42
Smoking, *n* (%)	5 (5.1%)	4 (5.8%)	4 (8.0%)	0.71
Alcohol consumption, *n* (%; yes)	76 (76.8%)	51 (73.4%)	40 (80.0%)	0.74
Diabetes, *n* (%)	3 (3.0%)	10 (14.5%)	3 (6.0%)	**0.019**
On treatment for systemic hypertension, *n* (%)	32 (32.3%)	28 (40.6%)	21 (42.0%)	0.40
On treatment for high cholesterol, *n* (%)	32 (32.3%)	29 (42.0%)	20 (40.0%)	0.39
Number of systemic pathologies, median (IQR)	1 (0–2)	2 (1–2)	1 (0–2)	0.20
Vasospasm, *n* (%)	31 (44.9%)	7 (10.1%)	3 (6.0%)	**<0.001**
Migraine, *n* (%)	16 (16.1%)	12 (17.4%)	4 (8.0%)	0.31
BMI, mean (±s.d.), kg m^−^^2^	24.2 (±3.7)	25.8 (±4.9)	28.0 (±5.5)	**<0.001**
Vitamin D, *n* (%)	18 (18.2%)	15 (21.7%)	12 (24.0%)	0.68
Thyroxine supplement, *n* (%)	6 (6.1%)	10 (14.5%)	1 (2.0%)	**0.047**
ACE inhibitors, *n* (%)	8 (8.1%)	14 (20.3%)	11 (22.0%)	**0.029**
ARB, *n* (%)	7 (7.1%)	1 (1.4%)	5 (10.0%)	0.11
BB, *n* (%)	16 (16.2%)	11 (15.9%)	12 (24.0%)	0.44
CCB, *n* (%)	18 (18.2%)	5 (7.2%)	7 (14.0%)	0.13
Diuretic, *n* (%)	8 (8.1%)	10 (14.5%)	7 (14.0%)	0.36
PGA drops, *n* (%)	60 (60.6%)	51 (73.9%)	N/A	0.10*
CAI drops, *n* (%)	37 (37.4%)	24 (34.8%)	N/A	0.86*
BB drops, *n* (%)	31 (31.3%)	25 (36.2%)	N/A	0.62*
a2A drops, *n* (%)	2 (2.0%)	6 (8.7%)	N/A	0.07*

Demographic and clinical characteristics among HTG, NTG and control groups were tested with ANOVA and chi-squared tests for continuous and categorical variables, respectively.

BB, β-blockers; CCB, calcium channel blockers; PGA, prostaglandin analogs; CAI, carbonic anhydrase inhibitors; a2A: α 2-agonists. Bold indicates *P* values < 0.05.

*Analysis done in two groups.

Extensive phenotypic information was collected for each participant (Supplementary Figs. 3 and 4). We next investigated the association of OCR with diagnostic category, demographic and clinical characteristics with univariable and multivariable linear regression models. We further analyzed those variables with a frequency of more than 15% and those detailed in the literature to have an impact on mitochondrial function. Covariates to include in the final multivariable models were selected with the least absolute shrinkage and selection operator (LASSO) regression^31^. Analyses were repeated on control subjects alone for comparison.

Results of the multivariable model for factors associated with basal OCR in all participants and in the subset of control subjects are illustrated in Fig. 1b and Supplementary Table 1. Older age was associated with higher basal OCR in the whole cohort (POAG and controls) but not in the subset of control subjects alone (Fig. 1b). There was no association between age and OCR in the univariable models for the whole cohort or in the subset of control subjects. Type 2 diabetes was associated with lower maximal OCR (estimate (standard error (s.e.): −52.0 (20.8) pmol min^−1^ per 100,000 cells, *P* = 0.016) and reserve capacity (estimate (s.e.): −48.8 (19.1) pmol min^−1^ per 100,000 cells, *P* = 0.014), in the subset of control subjects (Supplementary Fig. 5 and Supplementary Tables 2–4). None of the other covariates in any model was associated with mitochondrial function in control subjects. There was a nonsignificant association between systemic β-blocker use and basal OCR. To assess the combined effect of systemic and topical β-blockers, we ran a multivariable model combining β-blocker usage (topical and/or systemic). Results show no association with basal OCR (Supplementary Fig. 6).

To assess whether group differences in OCR were due to differences in PBMC subpopulations, lymphocyte and monocyte populations were measured in all participants at the time of cell counting using the MoxiGo II Flow Cytometer. Analysis of the two subpopulations was done using the FlowJo platform. There were no differences in the lymphocyte/monocyte ratio between groups. Additionally, gene expression levels were measured in a total of 30 randomly selected samples (10 from each group) using digital droplet polymerase chain reaction (ddPCR) and the following markers: CD56/NCAM1 for natural killer cells, CD14 for monocytes, CD9 for B cells, CD8A for T CD8 cells and CD4 for T CD4 cells. There were no differences in gene expression levels between control and patients with glaucoma, while basal OCR in this subset was significantly lower in the disease groups compared to controls (*P* < 0.001; Supplementary Fig. 7).

### The rate of VF progression is associated with mitochondrial respiration

VF progression rate and its association with mitochondrial respiration was analyzed in 229 eyes (NTG: 144 eyes, HTG: 85 eyes) of 139 patients. Table 2 shows the demographic and clinical characteristics of the cohort. The summary value for VF loss is the mean deviation (MD). The relationship between the rate of MD change and OCR was evaluated with linear mixed models with random slopes and random intercepts. In all models, the dependent variable was the MD value at each visit. Fixed effects were the follow-up time, covariates of interest and their interaction. The random slope term was the follow-up time to account for the fact that different eyes may have different rates of progression over time; the random intercept had a nested design with eye and patient IDs being the inner and outer level to account for within-eye (multiple tests from the same eye) and within-subject (both eyes of the same patient) correlations. Interactions between covariates and follow-up time modeled the variables’ effect on the progression rate. In addition to mitochondrial OCR, we included the following potential variables of interest: baseline age, central corneal thickness (CCT) and mean IOP over the VF observation period. Other OCR parameters (basal, ATP-linked and maximal OCR and reserve capacity) and IOP metrics (mean, peak, fluctuation and relative reduction) over the VF observation period were included in separate models as sensitivity analyses (Supplementary Figs. 8–11 and Supplementary Tables 5–11). All analyses were repeated on the HTG and NTG cohorts separately. All independent variables were standardized (that is, zero mean and unit s.d.) to facilitate the comparison of the association magnitude of different covariates by putting them on the same scale.

**Table 2:** Demographic and clinical characteristics of the subset of patients included in the VF analysis

Variable	Entire cohort	NTG	HTG
Eyes/patients, *n*	229/139	144/85	85/54
Baseline age, mean (±s.d.), years	63.7 (±10.4)	63.2 (±10.7)	64.5 (±10.0)
Sex, female/male, *n*	86/53	54/31	32/22
Laterality, right/left, *n*	118/111	74/70	44/41
CCT, mean (±s.d.), µ	539 (±42)	535 (±39)	545 (±46)
Baseline MD, median (IQR), dB	−3.97 (−1.86 to −8.04)	−4.94 (−1.96 to −9.54)	−2.80 (−1.09 to −6.18)
MD rate, mean (±s.d.), dB yr^−1^	−0.59 (±0.33)	−0.61 (±0.35)	−0.57 (±0.29)
Baseline IOP, median (IQR), mmHg*	15 (13–18)	14 (12–16)	18 (15–21)
Mean IOP, mean (±s.d.), mmHg*	14.9 (±2.8)	13.6 (±1.9)	17.0 (±2.7)
Peak IOP, median (IQR), mmHg*	19 (16–21)	17 (15–19)	22 (20–25)
s.d. IOP, median (IQR), mmHg*	2.2 (1.7–2.9)	2.0 (1.7–2.4)	2.6 (1.9–3.6)
Pretreatment IOP, median (IQR), mmHg	18 (15–22)	16 (14–18)	24 (22–28)
IOP reduction, median (IQR), %*	20.7 (8.3– 31.4)	17.1 (4.5– 23.7)	31.9 (20.1– 41.7)
Length of follow-up, median (IQR), years	5.7 (4.6–6.9)	5.0 (4.2–6.4)	6.4 (5.4–8.6)
Number of visual fields, median (IQR)	10 (8–12)	10 (9–12)	9 (8–11)

*The baseline and final IOP in the series corresponded to the date of the first and last VF included in the analyses.

**Fig. 2:**
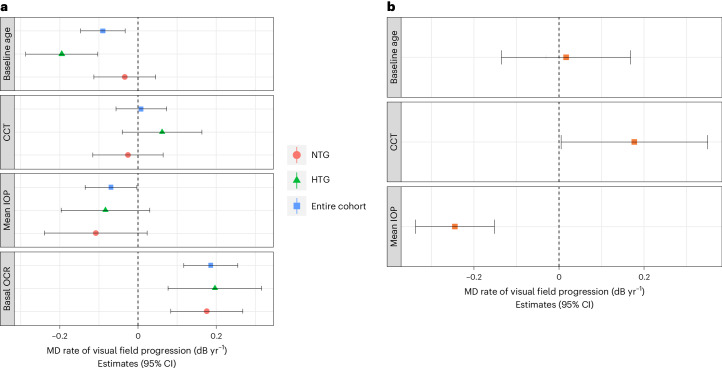
Association of mitochondrial respiration and IOP with VF progression. **a**, Forest plot shows the results of the basal OCR mixed effect model for factors associated with the MD VF progression, expressed as dB yr^−1^. The relationship between the rate of MD change and OCR was evaluated with linear mixed models with random slopes and random intercepts. Blue squares, green triangles and red circles represent standardized estimates, whereas horizontal bars represent their corresponding 95% CIs. All independent variables were standardized (that is, zero mean and unit s.d.). A total of 229 eyes (NTG: 144 eyes, HTG: 85 eyes) of 139 patients were included in this analysis. Older age was associated with faster rates of progression in the entire cohort (for a 10-year increase, estimate (s.e.): −0.09 (0.03) dB yr^−1^, *P* = 0.002). Higher mean IOP was associated with faster rates of progression in the entire cohort (for 1 s.d. (2.8 mmHg) increase, estimate (s.e.): −0.07 (0.03) dB yr^−1^, *P* =0.04). Lower basal OCR was associated with faster rates of progression in the entire cohort (for 1 s.d. (4.3 pmol min^−1^ per 100,000 cells) reduction, estimate (s.e.): 0.19 (0.04) dB yr^−1^, *P* < 0.001), NTG cohort (for 1 s.d. (4.1 pmol min^−1^ per 100,000 cells) reduction, estimate (s.e.): 0.18 (0.05) dB yr^−1^, *P* < 0.001) and HTG cohort (for 1 s.d. (4.0 pmol min^−1^ per 100,000 cells) reduction, estimate (s.e.): 0.20 (0.06) dB yr^−1^, *P* = 0.002). **b**, Forest plot shows the results of the mean IOP mixed effect model for factor associated with the MD VF progression, in the placebo arm of the UKGTS cohort. The relationship between the rate of MD change and IOP was evaluated with linear mixed models with random slopes and random intercepts. A total of 213 eyes were included in the analysis. Red squares represent standardized estimates, whereas horizontal bars represent their corresponding 95% CIs. All independent variables were standardized (that is, zero mean and unit s.d.) using the s.d. and mean of the parameters from the main paper to facilitate comparison. Higher mean IOP was significantly associated with faster VF progression (for 1 s.d. increase, estimate (s.e.): −0.25 (0.05) dB yr^−1^, *P* < 0.001), while thicker CCT with slower VF progression (for 1 s.d. increase, estimate (s.e.): 0.18 (0.09) dB yr^−1^, *P* = 0.046).

Overall, the mean (±s.d.) MD rate was −0.59 (0.33) dB yr^−1^. Lower basal OCR was significantly associated with faster rates of VF progression (Fig. 2a and Supplementary Table 5); estimate for 1 s.d. (4.3 pmol min^−1^ per 100,000 cells) difference (s.e.) was 0.19 (0.04) dB yr^−1^ (*P* < 0.001). The coefficients for the NTG and HTG cohorts were similar when analyzed separately (Fig. 2a and Supplementary Tables 6 and 7). The proportion of variance in the rate of progression explained by basal OCR was 13% (partial *R*^2^). Older age was associated with faster rates of progression in the whole cohort (for a 1 s.d. increase (10 years), estimate (s.e.): −0.09 (0.03) dB yr^−1^, *P* = 0.002) and in the HTG subset (for a 10-year increase, estimate (s.e.): −0.20 (0.05) dB yr^−1^, *P* < 0.001) but not in the NTG subset (for a 10-year increase, estimate (s.e.): −0.03 (0.04) dB yr^−1^, *P* = 0.40). Higher mean IOP was associated with faster rates of progression in the whole cohort (for 1 s.d. (2.8 mmHg) increase, estimate (s.e.): −0.07 (0.03) dB yr^−1^, *P* = 0.04) but not in the NTG and HTG groups separately. This equates to −0.03 (95% CI (−0.050 to −0.001)) dB yr^−1^ per 1 mmHg higher mean IOP. The proportion of variance explained by IOP was 1% (partial *R*^2^). In this cohort, a 1 s.d. difference in basal OCR was equivalent to a 7.6-mmHg IOP difference and a 21-year age difference.

To test the hypothesis that the association between OCR and VF progression rates may change as a function of IOP values, we ran additional models with a triple interaction term among follow-up time, basal OCR and mean IOP. The interaction term in the whole cohort was not significant (*P* = 0.97), suggesting that the association of mitochondrial dysfunction with disease progression is constant across the IOP spectrum (Supplementary Table 8). The interval from the end of the VF observation window and OCR measurement was a median (IQR) of 5.9 (1.7–9.5) years in patients who had undergone glaucoma surgery (50.7% of eyes) and 4.0 (2–18) days in patients who had not undergone glaucoma surgery. To test whether the association of OCR and VF progression rates was affected by whether patients had had glaucoma surgery or not (and the consequent time interval), we ran the analysis in two separate groups (surgery/no-surgery). The association between basal OCR and MD rate and the coefficients in both groups (surgery/no-surgery) were similar to those of the whole POAG cohort (Supplementary Tables 12 and 13). Furthermore, the interaction term between OCR and surgery status was not significant (*P* = 0.97), indicating that having had surgery was not associated with basal OCR differences (Supplementary Table 14). Taken together, these data support a hypothesis in which non-IOP factors (for example, genetic or nutritional) are associated with mitochondrial respiration and are biomarkers for visual function deterioration in patients with glaucoma.

### Association of IOP with VF progression

Glaucoma participants in our cohort were treated with IOP-lowering medication or surgery and this may alter the association between IOP and the rate of VF progression. Therefore, we looked at a separate untreated glaucoma cohort as a reference dataset—the placebo arm of the United Kingdom Glaucoma Treatment Study (UKGTS)^3,32^. We included eyes from the placebo group with ≥5 reliable (<15% false-positive responses) VFs. Demographic and clinical characteristics of this cohort are detailed in Supplementary Table 15. The median (IQR) MD progression rate was −0.23 (−0.73 to 0.11) dB yr^−1^. Only one eye per patient (worse baseline MD) was included in the analysis as per the UKGTS protocol. In all models, the dependent variable was the MD value at each visit; fixed effects were the follow-up time, covariates of interest, and their interaction, and the random slope term was the follow-up time. Results of the UKGTS multivariable model with standardized (by the SD of the clinic cohort) variables are illustrated in Fig. 2b. Higher mean IOP was significantly associated with faster VF progression—1 s.d. (2.8 mmHg) increase in IOP was associated with (estimate (s.e.)) −0.25 (0.05) dB yr^−1^ (*P* < 0.001) faster progression. A thicker central cornea was associated with slower VF progression (for 1 s.d. increase, estimate (s.e.): 0.18 (0.09) dB yr^−1^, *P* = 0.046). Baseline age was not associated with the rate of progression in this cohort (for 1 s.d. increase, estimate (s.e.): 0.017 (0.08), *P* = 0.83). The proportion of variance in the rate of VF progression explained by IOP in the placebo arm of the UKGTS was 16% (partial *R*^2^). In the clinical study, a reduction in basal OCR by 1 s.d. was associated with −0.19 dB yr^−1^ rate difference, an association size equivalent to 2.1 mmHg in the UKGTS placebo arm.

### Patients with glaucoma have lower blood cell NAD

**Fig. 3:**
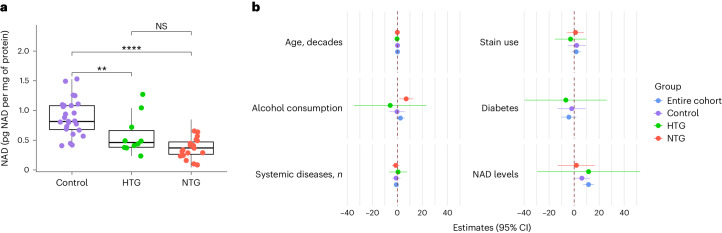
NAD levels in PBMCs and the association with basal OCR. **a**, Box plots and results of the Tukey HSD post hoc test for total cellular NAD levels in PBMC as a function of diagnostic category. Total NAD levels were measured in 54 (24.8%) subjects (25 controls, 10 HTG and 19 NTG). Total NAD levels followed the same pattern as basal OCR, with NTG patients having the lowest NAD levels (mean (s.d.): 0.4 (±0.2) pg NAD per mg of protein), followed by HTG (mean (s.d.): 0.6 (±0.3) pg NAD per mg of protein) and controls (mean (s.d.): 0.9 (±0.3) pg NAD per mg of protein). The box plots display the distribution of total NAD values within each diagnostic category. Each box plot represents the IQR of the data, with the horizontal line inside the box indicating the median value. The lower and upper bounds of the box represent the first and third quartiles, respectively. The ‘whiskers’ extend to the minimum and maximum values within 1.5 times the IQR from the lower and upper quartiles, respectively. One-way ANOVA with Tukey’s HSD test to control family type I errors for post hoc pairwise comparisons. No adjustment for multiple comparisons was made for all other tests. Specifically, NTG versus control comparison yielded a *P* value of <0.0001, HTG versus control comparison yielded a *P* value of 0.009 and HTG versus NTG comparison yielded a *P* value of 0.1. Significant change with *P < 0.05, **P < 0.01 and ****P < 0.0001. **b**, Forest plot showing the results of multivariable linear regression model for predicting basal OCR in the subset of participants who have total cellular NAD measurements (25 controls, 10 HTG and 19 NTG). Blue, red, green and purple circles represent β estimates from the multivariable regression model, and the horizontal bars represent the corresponding 95% CIs. The model predicts that an increase in total NAD levels of 1 pg NAD per mg of protein is associated with an increase in basal OCR of 11.6 pmol min^−1^ per 100,000 cells in the entire cohort (*P* < 0.001). The same trend, although not reaching statistical significance, was noted in each subgroup.

It has previously been reported that the NAD precursor, NAM, is low in sera of a cohort of patients with glaucoma^25^. To test the hypothesis that NAD levels themselves are lower in POAG (HTG and NTG), we measured total cellular NAD in the PBMC of 54 subjects (25 controls, 10 HTGs and 19 NTGs). The intra-assay CV between replicates was 9%. Cellular total NAD was statistically significantly different between the three groups (*P* < 0.001), with both NTG (*P* < 0.001) and HTG (*P* = 0.01) patients having significantly lower NAD levels compared to controls (Fig. 3a).

To test the hypothesis that NAD levels were associated with mitochondrial OCR, we ran multivariable linear regression models. Considering the smaller number of participants in this part of the study, covariates included in the model were based on the results of the multivariable models in the larger cohort. We chose to select covariates that were significantly associated with any of the OCR parameters (age, statin usage, number of systemic diseases, alcohol consumption and diabetes). Results of the multivariable model for basal OCR in the whole cohort of participants, and the subset of control subjects only, are illustrated in Fig. 3b and Supplementary Table 16. In the whole cohort, there was a significant association between higher cellular total NAD levels and higher basal OCR (estimate (s.e.): 11.6 (2.1) pmol min^−1^ per 100,000 cells per 1 pg NAD per mg of protein increase, *P* < 0.001). The proportion of variance in basal OCR in the multivariable model explained by total NAD levels was 16% (partial *R*^2^). In control samples, the association of higher NAD levels with higher basal OCR values did not achieve statistical significance at the nominal 5% level (estimate (s.e.): 6.5 (3.4) pmol min^−1^ per 100,000 cells per 1 unit of NAD increase, *P* = 0.07). The same trend, although not reaching statistical significance, was noted in the NTG and HTG subgroups (Fig. 3b). There were similar associations between total NAD and other OCR parameters, while other covariates were not associated with OCR in any of the models (Supplementary Fig. 12 and Supplementary Tables 17–19). There was no significant association between NAD levels and rates of VF MD progression (estimate (s.e.): 0.44 (0.37) dB yr^−1^ per 1 s.d. (0.36 pg NAD per mg of protein) NAD increase, *P* = 0.25), possibly due to the small sample size (the association between basal OCR and rates of VF MD progression is also not significant (estimate (s.e.): 0.14 (0.09) dB yr^−1^ per 1 s.d. basal OCR increase (4.3 pmol min^−1^ per 100,000 cells), *P* = 0.12) in this smaller subset of patients).

## Discussion

Elevated IOP and greater age are the major risk factors for glaucoma development and progression. While IOP remains the sole modifiable risk factor, increasing lines of evidence suggest that other factors contribute to glaucoma susceptibility and severity. Our study assessed the relationship between mitochondrial respiratory activity and NAD levels, examining their potential roles as biomarkers for glaucoma progression. The data presented here demonstrate significantly lower mitochondrial OCR in both HTG and NTG patients compared to age-similar controls, with the difference being more marked in the NTG cohort. These data also provide evidence of a strong, clinically relevant association between lower systemic mitochondrial function and faster glaucomatous VF progression in patients already treated by lowering IOP. Among various metabolites and pathways that may be implicated in mitochondrial function, lower NAD levels have been reported in various glaucoma models^15,33^. Our findings support this and demonstrate that total PBMC cellular NAD levels are lower in POAG patients and are strongly associated with lower mitochondrial respiratory function.

Initial reports of mitochondrial abnormalities in lymphocytes of patients with glaucoma^12^ have since been replicated by others^17^, including in our own study^34^. The role of mitochondria in glaucoma susceptibility is supported by ex vivo and animal model studies^15,35–40^ and genetic studies^41^. To understand the role of mitochondrial function in glaucoma, we ran multivariable models including various factors that may have an influence on mitochondrial function. Type 2 diabetes was the only covariate associated with lower (maximal) OCR in the control group, in agreement with previous studies^42^. NTG patients had the lowest mitochondrial function, compared to HTG and controls. IOP is a well-established risk factor in glaucoma; however, NTG patients develop glaucomatous optic neuropathy with IOP in the statistical normal range, indicating that IOP is only one factor for developing glaucoma^43^. The phenotype of the NTG neuropathy may exhibit subtle differences from that of HTG. Caprioli and Spaeth^44^ found VF defects in NTG to be substantially deeper and closer to fixation than in other types of glaucoma. While mitochondrial optic neuropathies such as Leber’s hereditary optic neuropathy (mitochondrial genome mutations) and dominant optic atrophy (mutations in nuclear genes encoding inner mitochondrial membrane proteins)^45^ have distinct phenotypes, there are some similarities with NTG, such as the more central VF loss. In fact, mutations in the optineurin and TBK1 genes (which mediate mitophagy) and polymorphisms in OPA1 (essential for mitochondrial fusion) account for a small proportion of NTG cases^46–49^. Given that mitochondrial function is lower in HTG than controls and lower in NTG than HTG, if future studies identify a causal link, it may be a risk factor across the range of IOP and more important at normal than at high IOP levels. The interaction term (IOP × OCR × time) in our statistical model was not significant, suggesting that the IOP risk and mitochondrial association are simply additive.

We evaluated the association between mitochondrial function and VF progression. The rate of VF progression is the most important clinical measurement of vision deterioration in glaucoma and helps predict the likelihood of visual disability and blindness^50^. The identification of biomarkers for faster deterioration, identifying patients most at risk of visual disability and blindness, would considerably improve glaucoma care efficiency and enable personalized medicine. Such biomarkers would enable risk stratification for more intensive monitoring, more appropriate setting of treatment goals (such as target IOP) and, should mitochondria-targeted therapies become available, selection of patients for targeted treatment. Our study identifies OCR as a new factor strongly associated with the rate of VF progression. Various clinical trials have demonstrated that reducing IOP slows down glaucoma progression in both HTG and NTG^4,5,51,52^. For instance, the Early Manifest Glaucoma Trial found that the risk of progression was reduced by 10% for each 1 mmHg IOP reduction^5^. In our study, we found the size of the association of basal OCR with the rate of VF loss in the whole cohort to be two and a half times that of IOP, when OCR and IOP were standardized to their respective SDs in this cohort. The proportion of variance explained by IOP (partial *R*^2^ = 0.01) was low. The explanation for the weak association of IOP with VF progression rate is that this is a clinical glaucoma cohort treated for IOP lowering. In clinical practice, IOP is lowered to a level considered sufficient to slow VF progression to prevent symptomatic vision loss in the patient’s lifetime^53^, and treatment is escalated in patients showing VF progression. Such clinical intervention alters the association between IOP and progression rate. Therefore, we examined the association between IOP and the rate of VF progression in a separate untreated glaucoma cohort (the placebo arms of the UKGTS) as a reference dataset. The proportion of variance in the rate of VF progression rate explained by IOP in the placebo arm of the UKGTS was about 16%. This compares to the proportion explained by basal OCR (13%) in the current study, once the contribution of IOP had been almost removed. The association of lower basal OCR with a faster rate of VF loss is clinically relevant, with 1 s.d. basal OCR in this study being equivalent to between 2.1 mmHg (in the UKGTS cohort) and 7.6 mmHg (in this study’s clinic cohort) higher mean IOP. Similarly, one s.d. lower basal OCR was equivalent to being nearly 21 years older, in terms of glaucoma progression risk in this dataset. This demonstrates that OCR is an important biomarker for progressive VF deterioration in both HTG and NTG.

Retinal NAD depletion in animal models of glaucoma and reduced NAM in plasma of POAG patients have been reported by others^15,25^. Our results suggest that NAD levels may be a mediator of reduced mitochondrial respiratory function and a biomarker for glaucoma progression. NAD is a central coenzyme in many metabolic processes. Depletion of NAD not only compromises ATP synthesis but also disrupts redox balance and signaling cascades, and low NAD drives Wallerian degeneration (axon degeneration)^54–58^. The low NAD levels in PBMC demonstrated in our study indicate possible systemic metabolic dysfunction outside the eye in patients with glaucoma. The systemic nature of the metabolic dysfunction is supported by similar findings in fibroblasts of a different cohort of NTG patients^59^. Although the precise mechanisms leading to mitochondrial dysfunction are still unclear, such dysfunction observed in glaucoma could be the result or cause of lower NAD levels. Considering the substantial energy demands of RGCs, their axons and the associated glial cells, coupled with the accumulating evidence from preclinical models, we hypothesize that the observed mitochondrial dysfunction in POAG is more than an epiphenomenon. Further study is, however, needed to prove causality. Taken together, these findings are consistent with the hypothesis, wherein non-IOP factors, such as genetic or nutritional influences, are associated with mitochondrial respiration and potentially serve as biomarkers for visual function deterioration in individuals with glaucoma.

There are some limitations in this study. Estimates of VF progression are, by the nature of VF testing, imprecise. This imprecision weakens the strength of the observed association between the rate of VF progression and OCR measurements and IOP, so that both OCR and IOP likely explain more of the variance in the true rates of progression than is apparent in the analysis. The OCR and NAD measurements were made after the VF observation window, by several years in those who had glaucoma surgery. However, an analysis of patients with a longer interval between measurements showed very similar results compared to those with a short interval, suggesting that the association of OCR and NAD measurements with progression risk is probably quite stable over time. Of 52 participants, 32 had both assays on the same day, while others were recruited coincidentally during routine appointments at Moorfields Eye Hospital based on their availability. This approach may underestimate the correlation between OCR and NAD due to variations in the time between measurements. The POAG patients in our study were recruited from clinics seeing patients at high risk of, or demonstrating, progression. This may overestimate the role of mitochondrial function as a biomarker in an unselected glaucoma population. Nevertheless, it quantifies the association of mitochondrial function with VF progression in the subset of patients of greatest importance—those more likely to lose vision from glaucoma. Mitochondrial function will be measured prospectively in an unselected POAG population in the NAMinG trial, providing information on the generalizability of the findings of this study. Recruitment was impeded by the COVID-19 pandemic and concluded before our intended sample size was achieved when the ethical permissions and the designated study period expired. The control subjects were recruited after most of the patients with glaucoma. Therefore, we assessed test–retest data in a 20% sample of the full dataset for signs of drift in OCR values over the study period (Supplementary Figs. 13 and 14). There was no evidence for a relationship between OCR values and the interval between the first and second tests over approximately 2½ years. Given the observational design of our study, we can only report associations and cannot infer causation; glaucoma may be, in part, a consequence of impaired mitochondrial function or NAD metabolism, vice versa or both. Mitochondrial respiratory function can be influenced by physical activity^60^ and levels of depression and anxiety^61^, which in turn may be influenced by the presence and severity of glaucoma^62^. While the lower OCR in NTG than HTG could support a causative role for mitochondrial function, the NTG patients had slightly greater VF loss than the HTG patients (median MD = −4.94 dB versus −2.80 dB, respectively), which could be associated with lower levels of exercise in the NTG patients. To address this, future research, such as the prospective NAMinG trial and TGNT (NCT05275738), will include variables such as physical activity and psychological well-being; the recruitment of newly diagnosed patients will also provide valuable insights into the generalizability of our findings. The OCR and NAD assays employed in this study have moderate repeatability and are too labor-intensive for clinical application. If the importance of these measurements in clinical routine becomes established, technological improvements should follow with benefits to test practicality, precision and cost.

In conclusion, this study provides evidence of the strong association between mitochondrial respiratory activity and the rate of disease progression in glaucoma. The findings have practical implications for clinical decision-making. Low OCR and/or NAD may identify patients at risk of faster progression, informing personalized treatment strategies and, if shown to be causative, offering the potential for mitochondria-targeted therapies.

## Methods

### Study design

This study was conducted in adherence with the tenets of the Declaration of Helsinki and was approved by the London—Surrey Borders and Health and Care Research Wales ethics committees. Participants were recruited between October 2018 and December 2021 after informed consent was taken from all participants. All participants underwent blood drawing. The study consisted of the following three parts (Supplementary Fig. 15): part 1, association of PBMC mitochondrial function with glaucoma diagnosis; part 2, association of PBMC mitochondrial OCR and the rate of VF progression and part 3, association of PBMC NAD levels with glaucoma diagnosis and OCR. The association between IOP and the rate of VF progression was evaluated in a reference cohort of patients without IOP-lowering treatment (to avoid the confounding effect of treatment escalation on the association)—the placebo arm of the UKGTS^3,32^. Supplementary Table 15 shows the main baseline demographic characteristics of this group.

The sample size calculation was based on a previous study that demonstrated lower complex-I-driven ATP synthesis in lymphocytes of NTG compared to patients with ocular hypertensive^34^. The effect size was 0.47 (ref. ^63^). Ninety-nine patients per group were needed to detect a difference with an α value of 0.05 and power = 0.80 (G*Power v.3.1.9.2). Recruitment was impeded by the COVID-19 pandemic and concluded before our intended sample size was achieved when the ethical permissions and the designated study period expired.

### Participants

A convenience sample of POAG patients was recruited from the glaucoma clinics at Moorfields Eye Hospital (London, UK) during their routine clinic appointments. Eligibility required a consultant ophthalmologist’s confirmation of the diagnosis of HTG (IOP ≥ 21 mmHg before treatment) or NTG (IOP ≤ 21 mmHg before treatment), open drainage angles on gonioscopy and absence of a secondary cause for glaucomatous optic neuropathy or nonglaucomatous cause for VF loss. POAG participants were under usual care of IOP-lowering eye drops (64% of eyes), laser trabeculoplasty (16% of eyes) and/or surgical IOP reduction (50.7% of eyes). Age-similar control participants were recruited from the cataract clinics at Moorfields Eye Hospital (London, UK)—eligibility required an ophthalmologist confirmation of the absence of glaucomatous optic neuropathy, no family history of glaucoma in a first-degree relative and IOP ≤21 mmHg.

Here 99 NTG, 69 HTG and 48 controls were recruited for part 1 of the study. To be eligible for part 2, participants were required to have had a minimum of six reliable (<15% false-positive responses) VFs over a minimum of 3 years before having had any form of glaucoma surgery (trabeculectomy/tube), if applicable. For eyes having had glaucoma surgery, follow-up was censored from the listing visit onward so that the observation window was immediately prior to glaucoma surgery. Also, 50.7% of eyes had undergone glaucoma surgery and 93% of eyes were on at least one IOP-lowering eye drop at the time of OCR measurement.

Exclusion criteria for all participants were given as follows: secondary (including pseudoexfoliation and pigmentary), angle closure and congenital glaucomas. In addition, participants were not eligible if they had any medical conditions, or were on any treatments, known to affect lymphocyte function (active hematological malignancy, infection at the time of the blood sampling and recent chemotherapy/radiotherapy) or mitochondrial function (amiodarone, tetracyclines, chloramphenicol, antiretroviral drugs).

Visual function was quantified by standard automated perimetry with the Humphrey Field Analyzer (Carl Zeiss Meditec, Inc.). We recorded the VF MD from age-matched normative values from 24-2 Swedish Interactive Thresholding Algorithm (SITA) standard tests, from both eyes. We also recorded the IOPs (Goldmann applanation tonometry (GAT)) of both eyes during the period for which VF data were collected. Extensive phenotypic data were collected for each participant, including information on general and ocular health (Tables 1 and 2).

An untreated reference cohort was taken from the UKGTS, a randomized, multicenter, triple-masked, parallel-group, placebo-controlled trial^3,32^. The trial consisted of 516 participants randomized (1:1) to either latanoprost 0.005% or placebo, scheduled to have 16 24-2 Humphrey VFs over 24 months. In our analysis, we included only those eyes that had a minimum of five reliable VFs over the observation period. VFs were also SITA standard and IOP was measured with GAT. Variables included in the model were mean IOP over the observation period, CCT and baseline age.

Experimental conditions and timings were similar to all participants, therefore, maintaining consistency across the groups.

Ethics approval was obtained from the London—Surrey Borders Research Ethics Committee and HRA and Gwasanaeth Moeseg Ymchwil Research Ethics Service for the participants recruited in the study, and by Moorfields and Whittington Research Ethics Committee for the UKGTS participants.

### Isolation of PBMCs

Venous blood was sampled in 10.0 ml Becton Dickinson (BD) Vacutainer blood collection tubes containing ethylenediamintetraacetic acid (EDTA; BD Biosciences). PBMCs were isolated from freshly drawn blood within 3 h following venipuncture and were used immediately for OCR and total NAD measurements. Whole blood was mixed with 10 ml 1× PBS on a 1:1 ratio, and then slowly pipetted down a 50 ml falcon over 15 ml of Lymphoprep (StemCell Technologies, # 07801). Tubes were centrifuged at 1,300*g* for 26 min with no brake at room temperature. After centrifugation, 4 ml of the cloudy PBMC band was collected using a sterile transfer pipette and added to two 15 ml tubes (2 ml in each) and topped up with 6 ml PBS. The capped 15 ml tubes were mixed by inversion and centrifuged at 800*g* for 10 min at room temperature. The supernatant was carefully aspirated, and the cell pellets were resuspended in 8 ml of PBS and centrifuged for a subsequent 10 min at 800*g* at room temperature. Following this centrifugation step, the supernatant was again carefully aspirated without disturbing the cell pellet, and PBMCs were resuspended in 0.8 ml of complete Seahorse media warmed at 37 °C.

### Application of Seahorse XFe24 to measure oxygen consumption in PBMC

The Mito Stress assay provides the estimation of different bioenergetic measures by monitoring the OCRs of living cells through the addition of various inhibitors of OXPHOS. OCR was quantified using the XFe24 Analyzer (Agilent Technologies) according to the manufacturer’s instructions. This technique allows real-time measurements of OCR in living cells. Cells were seeded at a density of 8 × 10^5^ cells per well, with four technical repeats per participant. One day before the assay, XFe24 Sensor Cartridge (Agilent Technologies) was hydrated overnight at 37 °C in a non-CO_2_ incubator to equilibrate. On the day of the assay, PBMCs were suspended in 0.8 ml of prewarmed (37 °C) Seahorse Base media (Seahorse Bioscience, Agilent Technologies, 102353-100) supplemented with 10 mM glucose, 1 mM sodium pyruvate, 2 mM glutamine final, adjusted to pH 7.4. Cells were counted using the Orflo MoxiGo II flow cytometer and diluted to 8 × 10^6^ per ml. Here 100 µl of cell suspension was transferred into each well of a Seahorse XFe24 plate, precoated with Corning Cell-Tak Cell and Tissue Adhesive (Sigma-Aldrich, CLS354240-1EA), and the plate was centrifuged for 2 min at 200*g*, brake off to ensure cells were fully attached. The plate was transferred in a 37 °C non-CO_2_ incubator for 30 min, following which, each well was topped up to a final volume of 500 ml with complete Seahorse Base media and transferred in a 37 °C non-CO_2_ incubator for further 30 min. OCRs were monitored through sequential injections of oligomycin (1.5 μM), carbonylcyanide-4-trifluoromethoxyphenylhydrazone (FCCP, 1.5 μM), and antimycin A/rotenone (1.6 μM/16 μM). The various parameters of the mitochondrial respiration were calculated as per the manufacturer’s protocol^64^, and results were expressed as pmol min^−1^ per 100,000 cells.

### Cellular total NAD content assay

Cellular total (NAD^+^ + NADH) NAD was measured in fresh PBMC in a convenience sample of 52 participants from the cohort of 218 subjects (25 control, 10 HTG and 17 NTG). The median (IQR) number of days between OCR and NAD assays was 0 (0–123) days (Supplementary Fig. 16). Total NAD was quantified by a luciferase assay provided in the NAD^+^/NADH-Glo Assay kit (Promega, G9071). Luminescence was recorded at 20 min after the addition of the NAD/NADH-Glo Detection Reagent using Cytation1 Imaging plate reader (Biotek Instruments Inc., Agilent), with the Gen5 v3.05.11 microplate reader and imaging software. Following several titrations, the minimum and maximum amount of total cellular protein required to be in the linear range of the assay were 2.1–39 µg (corresponding roughly to 150,000–4,000,000 PBMC). PBMCs were washed in ice-cold PBS and centrifuged at 13,000*g* for 10 min at 4 °C. Supernatant was discarded and the cell pellet was suspended in 200 µl ice-cold PBS. A volume of 30 µl of cell suspension was transferred in a 96-well, white-walled plate, followed by 30 μl of NAD/NADH-Glo Detection Reagent. There was a minimum of two technical repeats for each participant. To ensure recordings were in the linear range of the assay, serial dilutions were performed. Technical repeats with protein levels outside the linear range and those with fluctuating results over time were excluded from the analysis. Cellular total NAD levels were normalized to protein content and expressed as pg NAD per mg of protein.

### RNA extraction

Total RNA including small RNAs was isolated using the Monarch Total RNA Miniprep Kit (New England Biolabs), according to the manufacturer’s recommendations, treated with DNase I, and eluted with 50 μl of diethyl pyrocarbonate-treated water. RNA quantity and quality assessment were done using the NanoDrop (NanoDrop Technologies, ND-1000).

### Gene expression probes

ddPCR GEX Assay: ACTB, Cy5.5 (Bio-Rad, 12005585)

ddPCR GEX Assay: NCAM1 (CD56), Cy5 (Bio-Rad, 12005582)

ddPCR GEX Assay: CD14, FAM (Bio-Rad, 10031252)

ddPCR GEX Assay: CD19, Cy5 (Bio-Rad, 12005582)

ddPCR GEX Assay: CD8A, HEX (Bio-Rad, 10031255)

ddPCR GEX Assay: CD4, FAM (Bio-Rad, 10031252)

### Digital droplet polymerase chain reaction

This experiment was conducted on 30 PBMC samples, selected at random (*n* = 10, per group). Results were analyzed using the QX ONE Software 1.4 Standard Edition (Bio-Rad). The researcher performing the assay and analysis was blinded to the sample ID/group. The experiment was done using the One-Step RT-ddPCR advanced Kit (Bio-Rad, 1864021) as per the manufacturer protocol.

### Flow cytometry

Lymphocyte and monocyte populations were measured in all participants at the time of cell counting—using the MoxiGo II Flow Cytometer featuring an optical detection range of 561 nm per LP. Analysis of the two subpopulations was done using the FlowJo platform. To establish gating parameters for monocyte and lymphocyte populations based on cell size, PBMCs were subjected to staining with REAfinity antibodies—CD19, CD3, CD14 (as a positive control) and IgG1 (as a negative control). The staining process was carried out in a buffer solution comprising PBS with a pH of 7.2, 0.5% bovine serum albumin and 2 mM EDTA, maintained on ice. After lymphoprep, cells were suspended at 1 million cells per ml in 1 ml PBS, followed by centrifugation, discarding the supernatant, and resuspending cells in 98 μl of the prepared buffer. A volume of 2 μl of each antibody (separately) was then added, and the mixture was incubated in the dark at 2−8 °C for 10 min—antibody dilution factor 1:50. Subsequent steps involved washing (1 ml of the preprepared buffer), centrifugation (300*g* for 10 min) and suspension in 1 ml PBS. Further dilution for flow cytometry was done (10 μl of the cell suspension in 190 μl PBS), and 60 μl of the diluted suspension was loaded into a Flow cytometry cartridge for cell analysis. This experiment allowed for the identification of gates for monocytes and lymphocytes based on cell size (Supplementary Fig. 17). Experiment was done in all participants. Experiment was failed for 1 NTG and 6 HTG participants. A total number of participants were given as follows: control 50, HTG 63 and NTG 98.

On the day of the Seahorse assay, cells were suspended in complete Seahorse media to achieve a concentration of 8 million cells per ml as described previously. Subsequently, 37.5 µl of this cell suspension was diluted in 1 ml of PBS, ensuring cell numbers fell within the detection range of the flow cytometer. Using 60 µl of this diluted cell suspension, the monocyte and lymphocyte populations were counted within the gates set in the previous section.

### Statistical analysis

We reported mean (±s.d.) and median (IQR) for continuous variables and frequencies and proportions for discrete variables. We compared demographic and clinical characteristics among HTG, NTG and control groups with ANOVA and chi-squared tests for continuous and categorical variables, respectively.

We investigated associations between various OCR metrics and demographic and clinical characteristics of enrolled subjects with univariable and multivariable linear regression models. We ran separate models for each OCR metric (dependent variable). Candidate covariates included age at the time of blood sample taking, sex, diagnostic status (that is, control, HTG and NTG), smoking status, alcohol consumption, diabetes, systemic hypertension, hypercholesterolemia, migraine, number of systemic illnesses, body mass index (BMI), vasospasm, vitamin D supplementation, thyroid disease and use of the following: angiotensin converting-enzyme (ACE) inhibitors, angiotensin receptor blocker (ARB), systemic β-blocker, calcium channel blocker and/or diuretics. Covariates included in each multivariable model were selected with the LASSO regression; LASSO is a form of penalized linear regression, which reduces some coefficients’ magnitude and shrinks other variables to zero; nonzero coefficients are kept in the model^31^. Analyses were also repeated on control subjects (after excluding glaucoma patients) for comparison. Given the extensive pool of potential covariates, we opted to include in the LASSO regression analysis those variables with a prevalence of 15% or higher. We also decided to include in the final model those variables that had a prevalence of less than 15% such as type 2 diabetes, or were not significant following LASSO regression, but are well-known through literature to impact mitochondrial function. Supplementary Figs. 11 and 12 show the distribution of general health conditions and medication usage across different participant types.

We used a one-way ANOVA with post hoc Tukey’s HSD test for multiple comparisons, to investigate whether cellular total NAD levels were different between groups (control, HTG and NTG). When assessing the relationship between total NAD levels and different parameters of OCR, we used a multivariable model. Considering the smaller number of participants in this part of the study, we selected the following covariates based on the results of the multivariable models in part 1: age, statin usage, number of systemic diseases, alcohol consumption and diabetes.

All tests were two-tailed, and *P* < 0.05 were considered statistically significant. We reported point estimates along with s.e. and *P* values for regression models. All analyses were performed with the statistical software R (R Foundation for Statistical Computing, Vienna, Austria). The relationship between the rate of MD change and IOP was evaluated with linear mixed models with random slopes and random intercepts. To evaluate the contribution of OCR within the multivariable model to VF progression rates, we calculated the coefficient of partial determination (partial *R*^2^) of the interaction term between OCR and time. The partial *R*^2^ measures the incremental contribution of one regression term when all other variables are included in the model. To calculate the partial *R*^2^, we constructed the following two multivariable linear mixed models: a full model and a reduced model. Both models had the same specifications, except that the reduced model lacked the OCR term. We then computed the sum of squares error (SSE) for the random effect in both models and calculated the partial *R*^2^ using the following formula:

$${\rm{partial}}\,{{{R}}}^{2}=\frac{{\rm{SSE}}\,({\rm{reduced}}\,{\rm{model}})-{\rm{SSE}}\,({\rm{full}}\,{\rm{model}})}{{\rm{SSE}}\,({\rm{reduced}}\,{\rm{model}})}$$

For the placebo arm of the UKGTS, we also calculated the partial *R*^2^ for mean IOP. We ran a full multivariable linear mixed model with random intercept and random slope having the same covariates (that is, baseline age, CCT and mean IOP) as this study cohort, and their interaction with follow-up time, with the exception of OCR, the measurement of which was not part of the UKGTS protocol. We then ran a reduced model that lacked the mean IOP term and calculate the partial *R*^2^ using the formula detailed above.

### Reporting summary

Further information on research design is available in the Nature Portfolio Reporting Summary linked to this article.

## Online content

Any methods, additional references, Nature Portfolio reporting summaries, source data, extended data, supplementary information, acknowledgements, peer review information; details of author contributions and competing interests; and statements of data and code availability are available at 10.1038/s41591-024-03068-6.

### Supplementary information

Supplementary Information Supplementary Figs. 1–17 and Supplementary Tables 1–19.

Reporting Summary

## Data Availability

All data generated or analyzed during this study are publicly available at the UCL Research Data Repository (https://figshare.com/s/145fd5cfbd9eaadeb857).
