# Supplementary material for: Peripheral blood mononuclear cell respiratory function is associated with progressive glaucomatous vision loss
Source: Nat Med. 2024 Jun 17;30(8):2362–70. doi: 10.1038/s41591-024-03068-6 (PMC11333286; doi:10.1038/s41591-024-03068-6)
Supplement: Supplementary file 1 — Supplementary Figs. 1–17 and Supplementary Tables 1–19. [file 41591_2024_3068_MOESM1_ESM.pdf]

nature medicine

Supplementary information https://doi.org/10.1038/s41591-024-03068-6

Peripheral blood mononuclear cell respiratory function is associated with progressive glaucomatous vision loss

In the format provided by the authors and unedited

Supplementary Figure 1

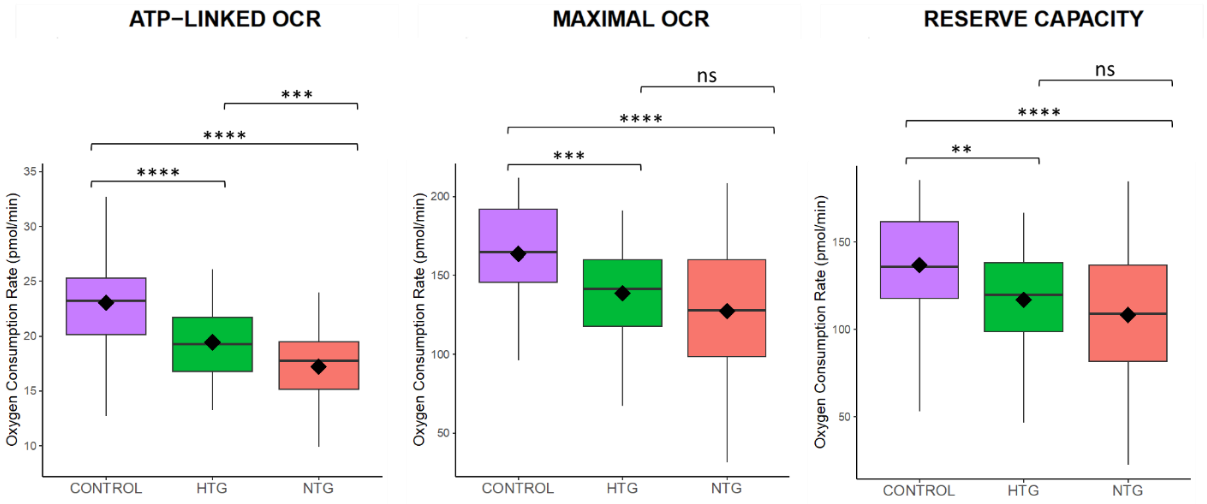

Sup. Fig. 1. Boxplots and results of the Tukey HSD post-hoc test for various oxygen consumption rate parameters in PBMC as a function of diagnostic category. OCR was measured using the XFe24 Analyzer in two hundred and eighteen participants (50 controls, 69 HTG, 99 NTG). The box plots display the distribution of OCR values within each diagnostic category. Each box plot represents the interquartile range (IQR) of the data, with the horizontal line inside the box indicating the median value. The lower and upper bounds of the box represent the first and third quartiles, respectively. The 'whiskers' extend to the minimum and maximum values within 1.5 times the IQR from the lower and upper quartiles, respectively. One-way ANOVA with Tukey’s HSD Test to control family type I errors for post-hoc pairwise comparisons. No adjustment for multiple comparisons were made for all other tests. Significant change with P value < 0.05 marked with *, P value < 0.01 marked with **, P value < 0.001 marked with ***, P value < 0.0001 marked with ****.

Supplementary Figure 2

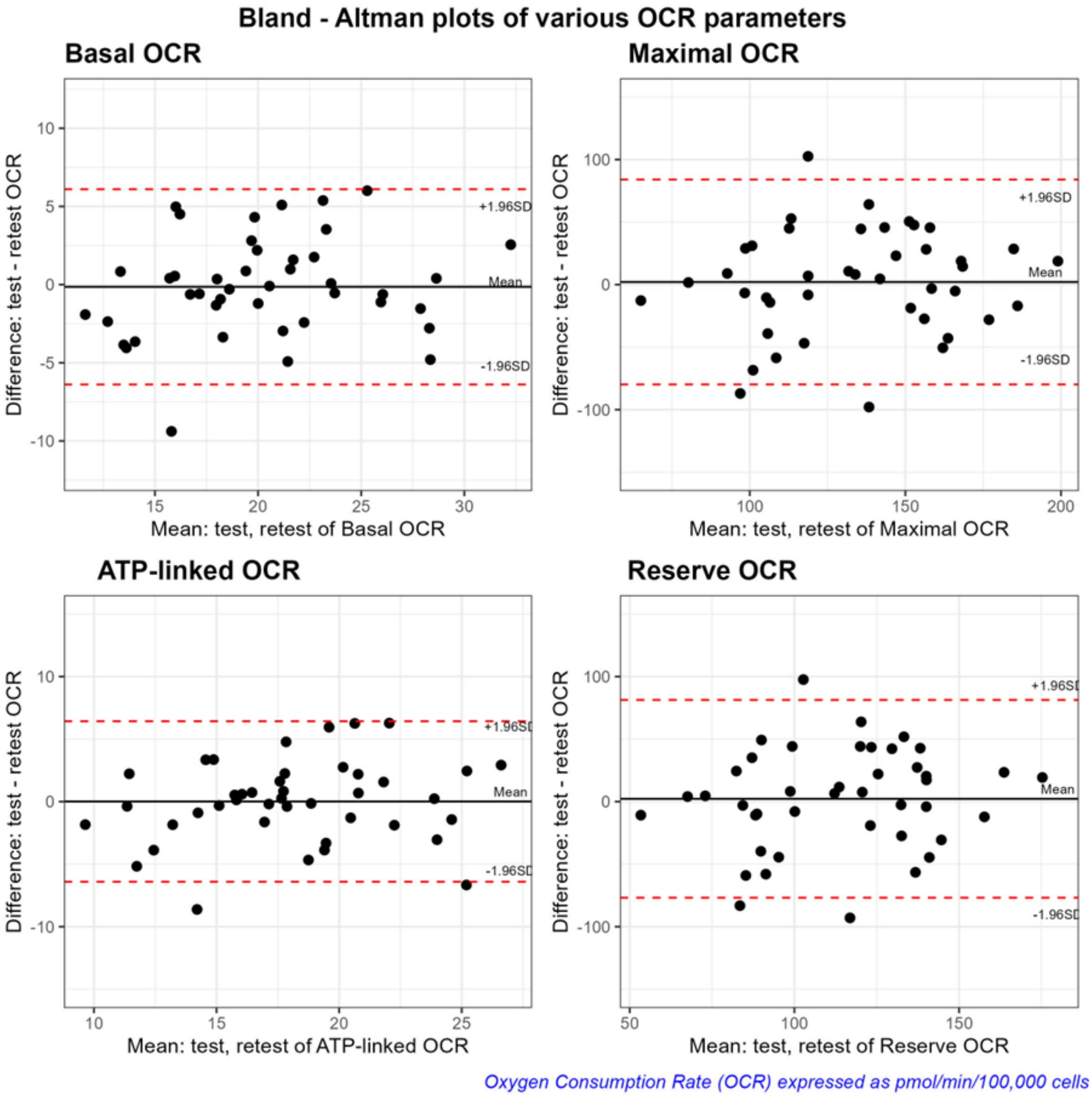

Sup. Fig. 2. Bland-Altman plots showing agreement between test - retest of the various mitochondrial oxygen consumption parameters measured by the Seahorse XFe24 Analyzer.

Supplementary Figure 3

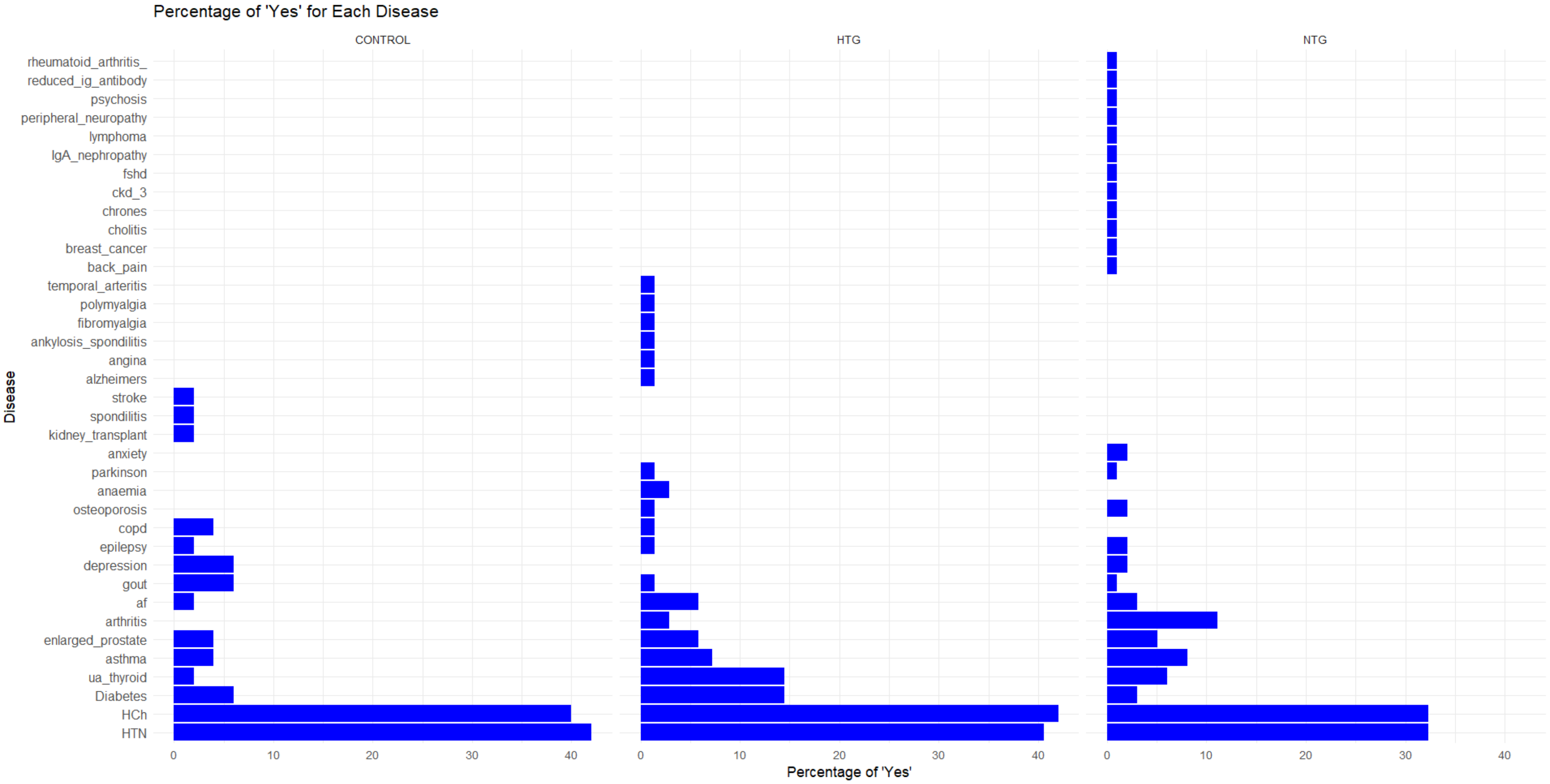

Supp. Fig. 3. Distribution of general health conditions in the study population, presented as the percentage of individuals with affirmative responses in each group.

Supplementary Figure 4

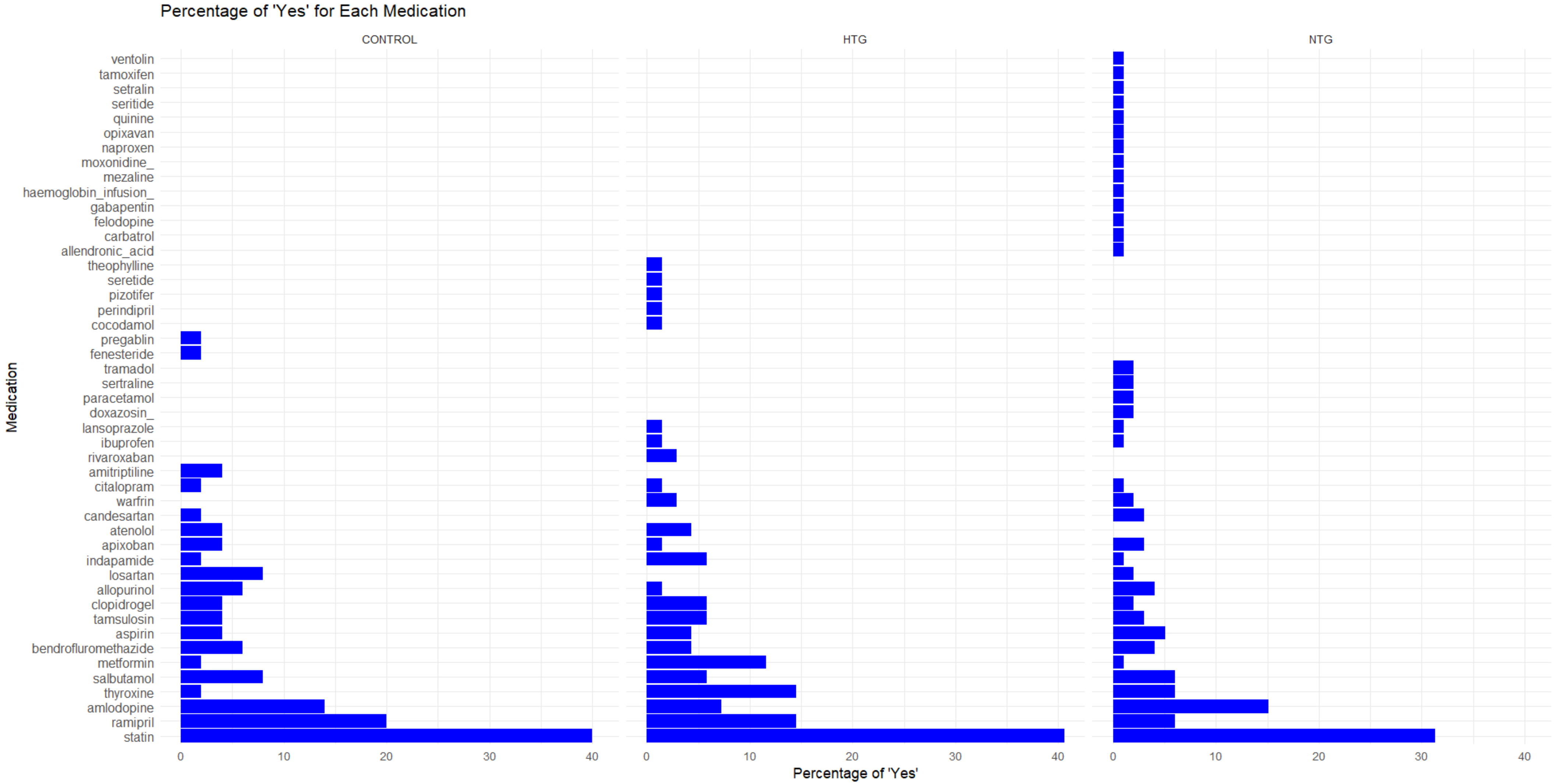

Supp. Fig. 4. Distribution of medication usage across different participant types in the study population, presented as the percentage of individuals with positive responses in each group

Supplementary Figure 5

ATP-linked OCR Multivariable Model

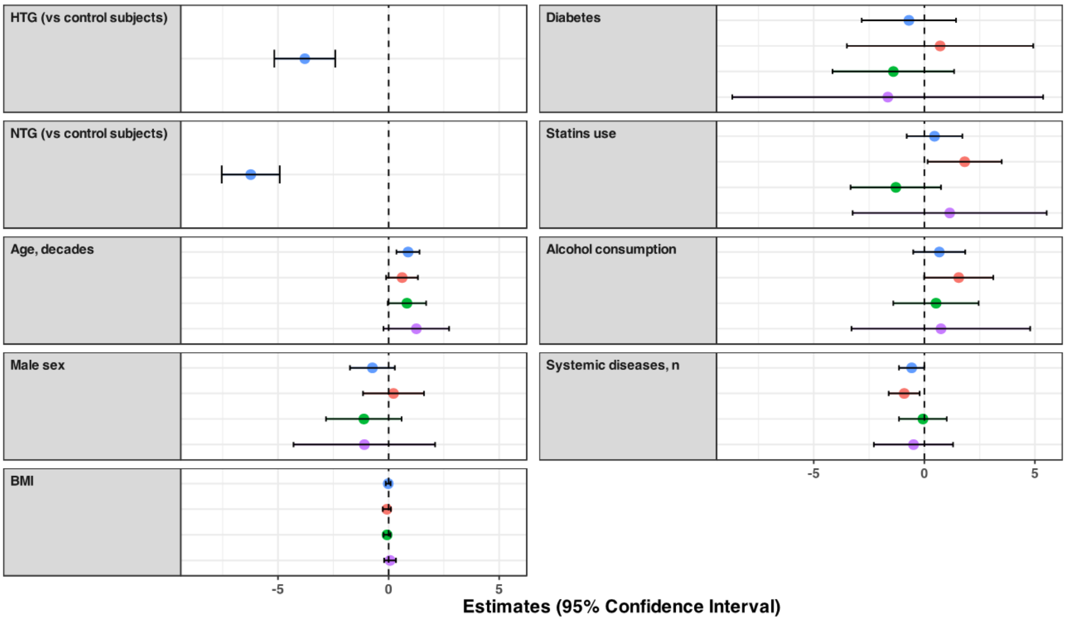

Maximal OCR Multivariable Model

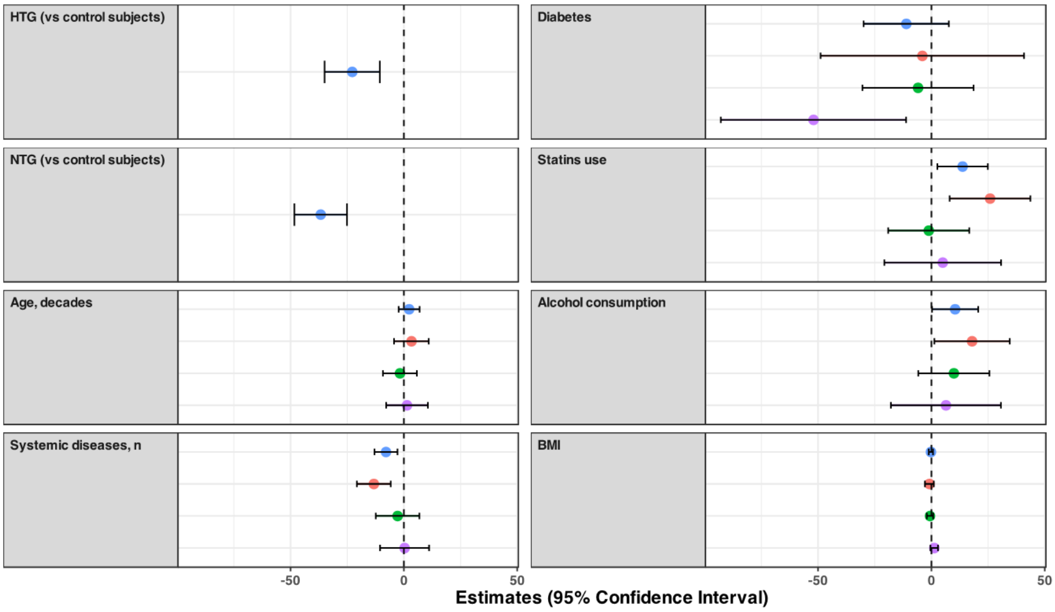

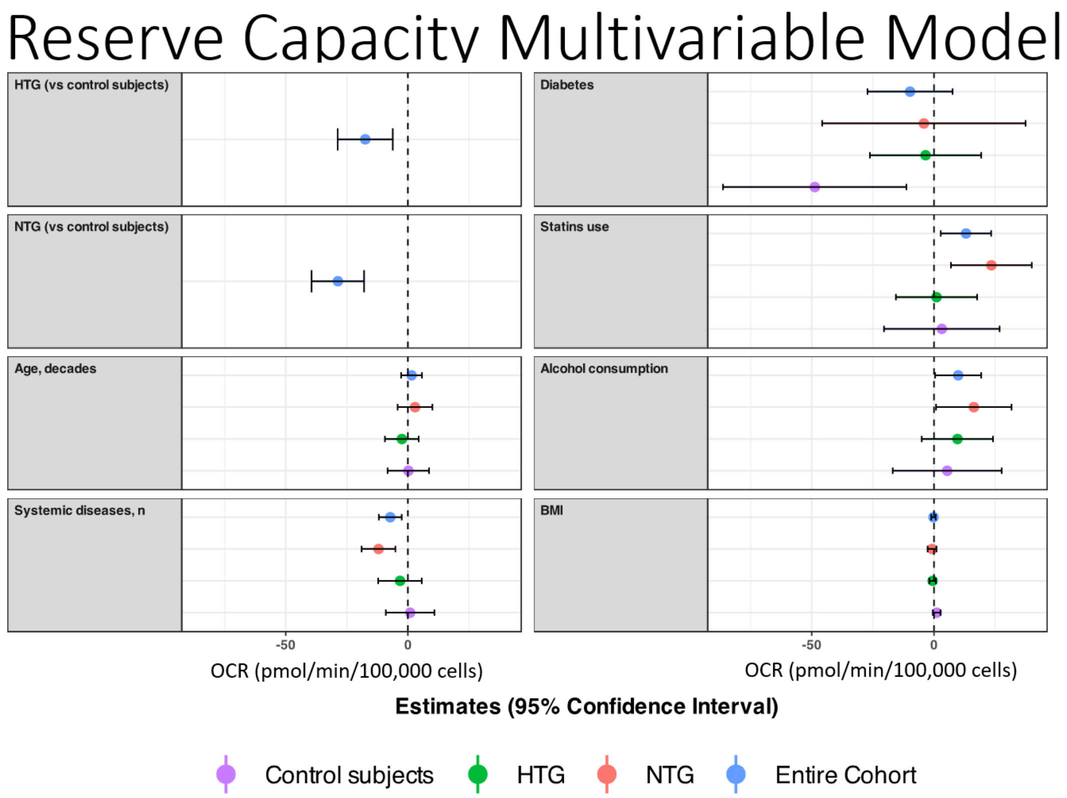

Sup. Fig. 5. Forest Plot showing the results of the multivariable model for factors associated with various OCR parameters in two hundred and eighteen participants (50 controls, 69 HTG, 99 NTG). Blue, red, green, and purple circles represent beta estimates from the multivariable regression model, and the horizontal bars represent the corresponding 95% confidence intervals.

Supplementary Figure 6

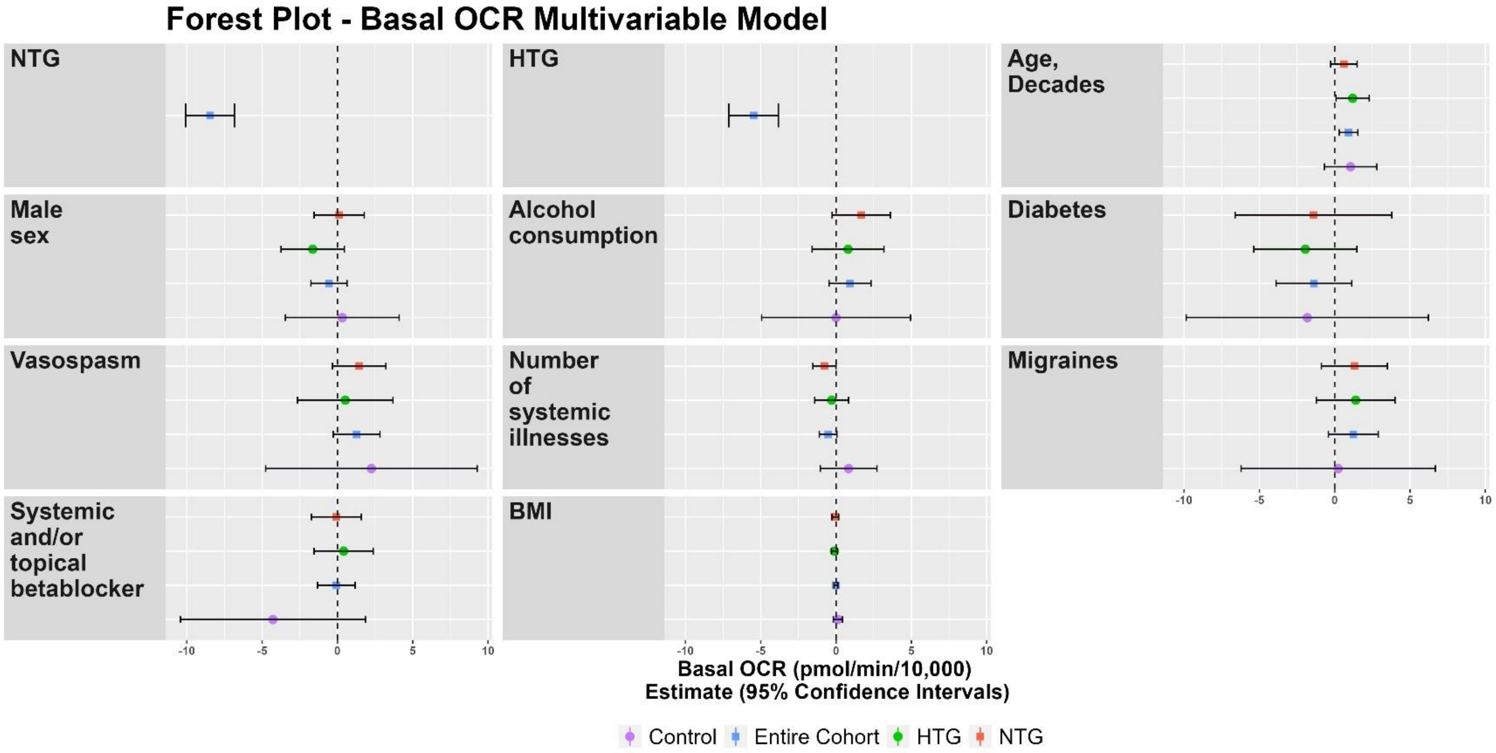

Sup. Fig. 6. Forest Plot showing the results of the multivariable model for factors associated with various OCR parameters. Analysis conducted in 50 controls, 69 HTG and 99 NTG participants. In this model, systemic and topical Beta-blockers have been combined to assess the combined effect. Blue, red, green and purple circles represent beta estimates from the multivariable regression model, whereas horizontal bars represent their corresponding 95% confidence intervals

Supplementary Figure 7

A

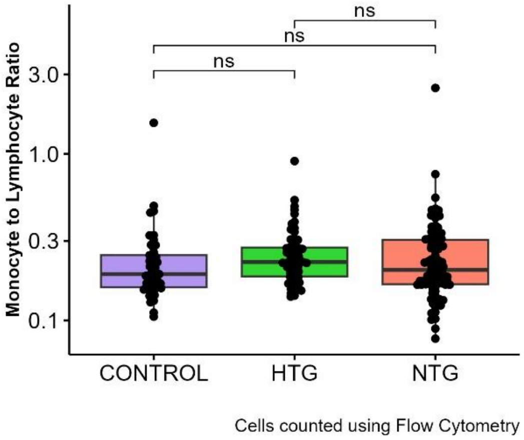

B

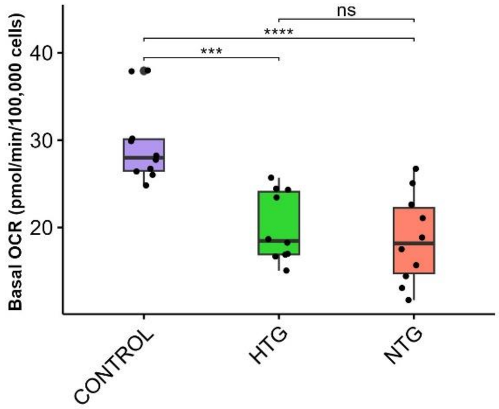

C

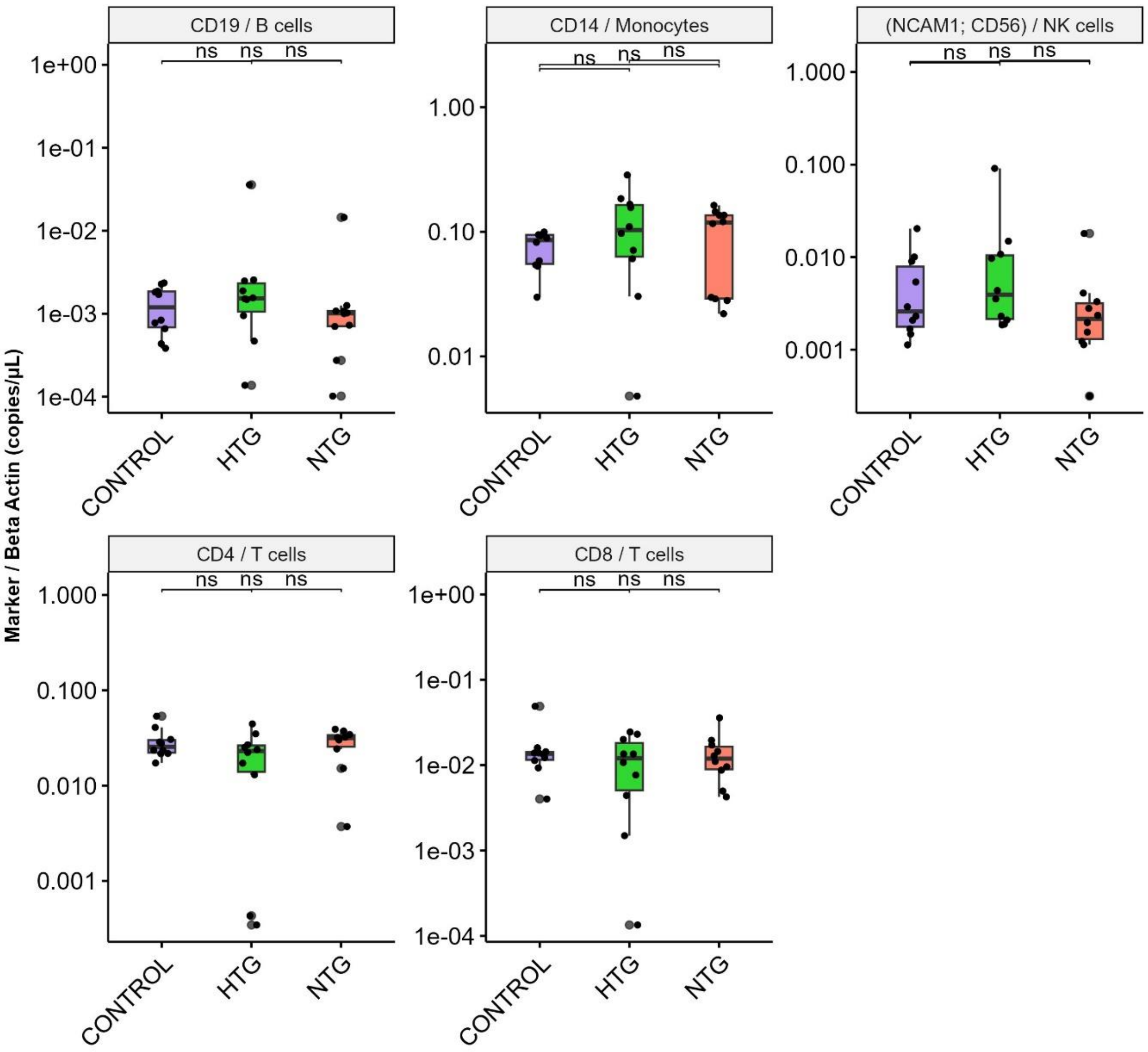

Sup. Fig. 7. **A**. Lymphocyte and Monocyte populations were measured using the MoxiGo II Flow Cytometer, at the time of cell counting prior to the Seahorse assay and expressed as a ratio of Monocyte/Lymphocyte (50 controls, 63 HTG, 98 NTG). Analysis of the two subpopulations was done using the FlowJo platform. There was no difference in the ratio of Monocyte to Lymphocyte between the groups. **B**. Basal OCR in a randomly selected subset of 30 participants (n=10 per group). Both NTG and HTG participants have significantly lower OCR than controls. **C**. Gene expression levels in the same 30 participants, measured using digital droplet polymerase chain reaction (ddPCR) and the following markers: CD56/NCAM1 for Natural Killer cells, CD14 for monocytes, CD9 for B cells, CD8A for T CD8 cells, and CD4 for T CD4 cells. Data was normalised to expression levels of Beta Actin. There were no differences in gene expression levels of various PBMC subpopulations between groups. The box plots display the distribution of values within each diagnostic category. Each box plot represents the interquartile range (IQR) of the data, with the horizontal line inside the box indicating the median value. The lower and upper bounds of the box represent the first and third quartiles, respectively. The 'whiskers' extend to the minimum and maximum values within 1.5 times the IQR from the lower and upper quartiles, respectively. One-way ANOVA with Tukey's HSD Test to control family type I errors for post-hoc pairwise comparisons. No adjustment for multiple comparisons were made for all other tests. Significant change with P value < 0.05 marked with *, P value < 0.01 marked with **, P value < 0.0001 marked with ****.

Supplementary Figure 8

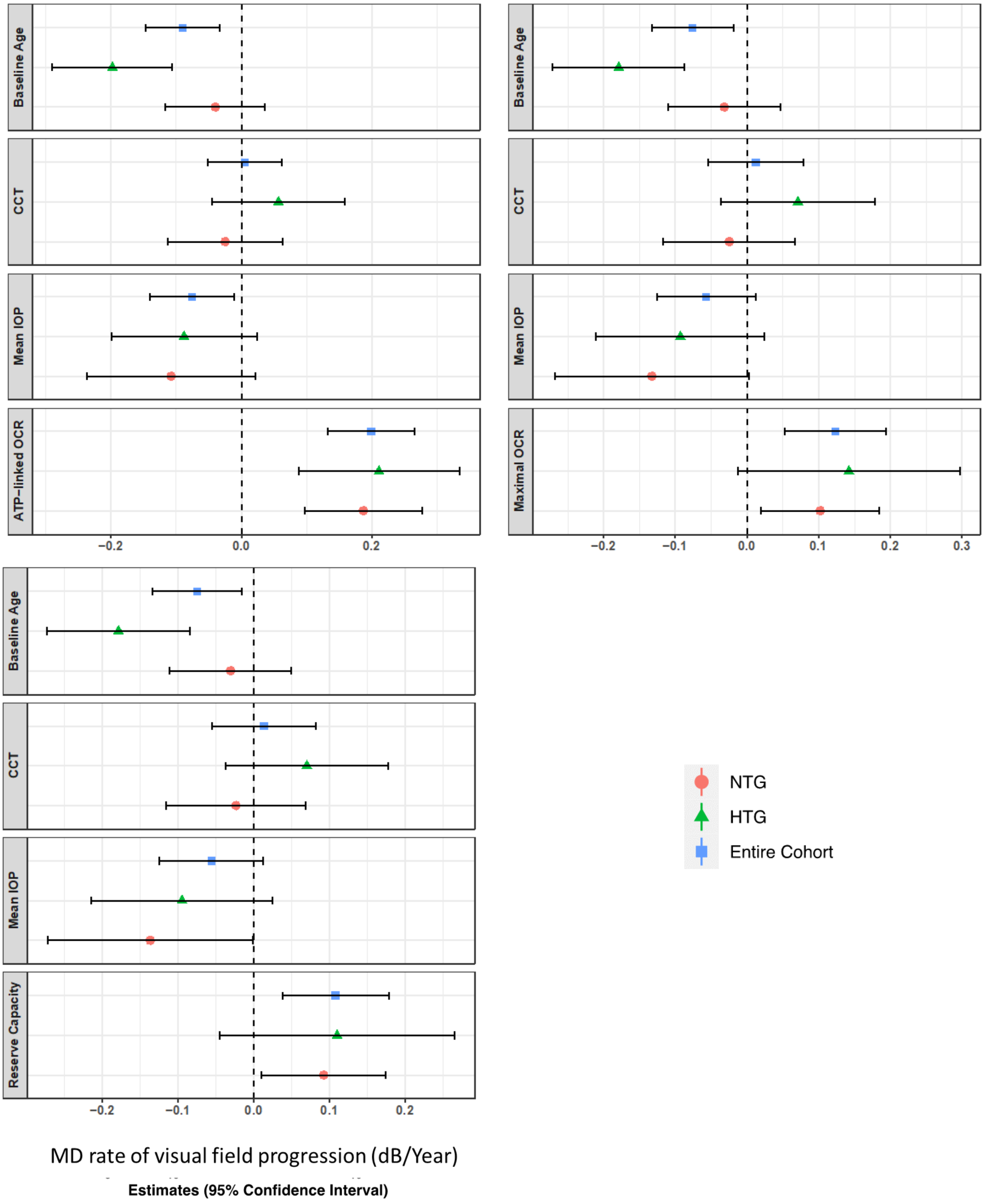

Supplementary Figure 9

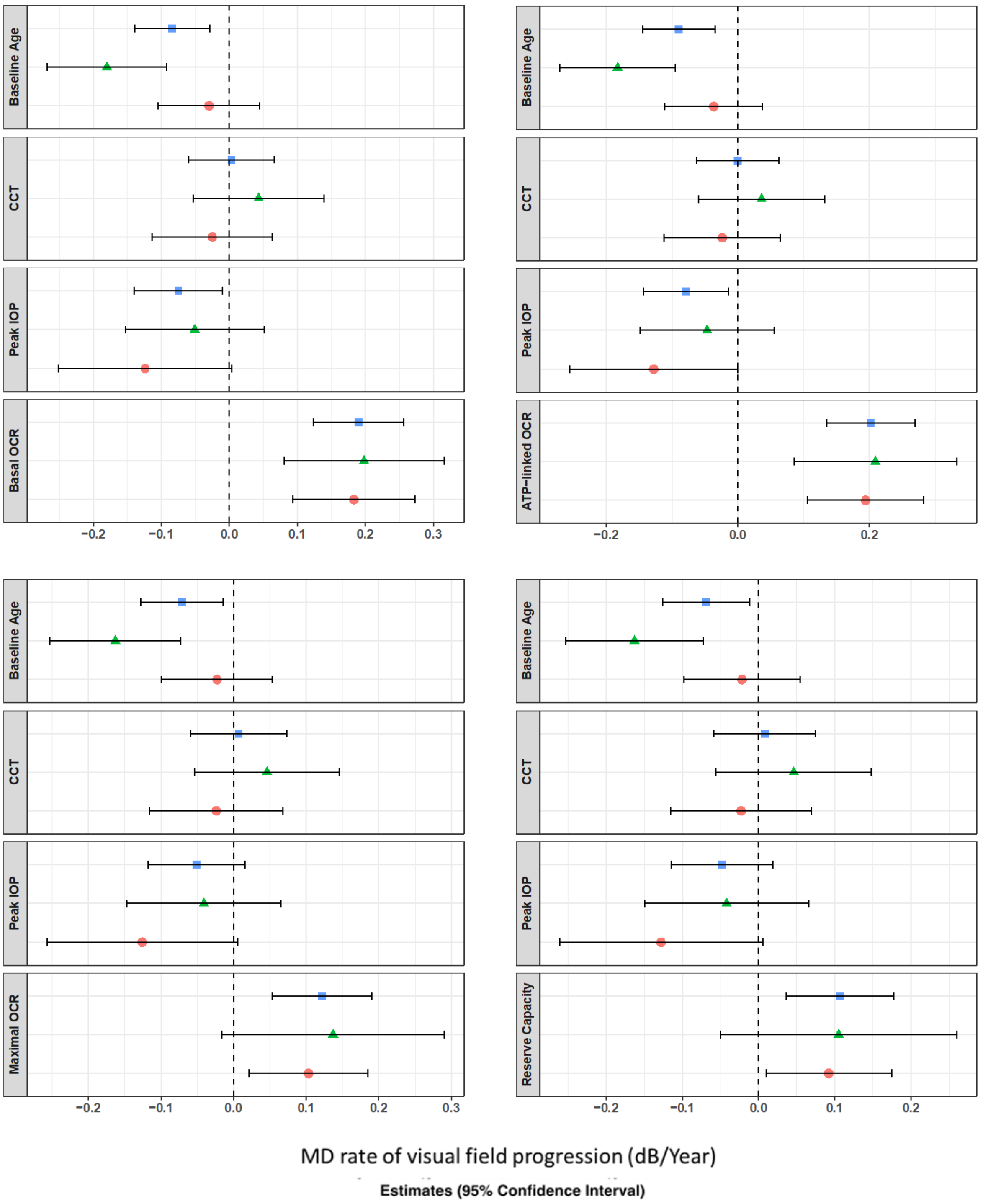

Supplementary Figure 10

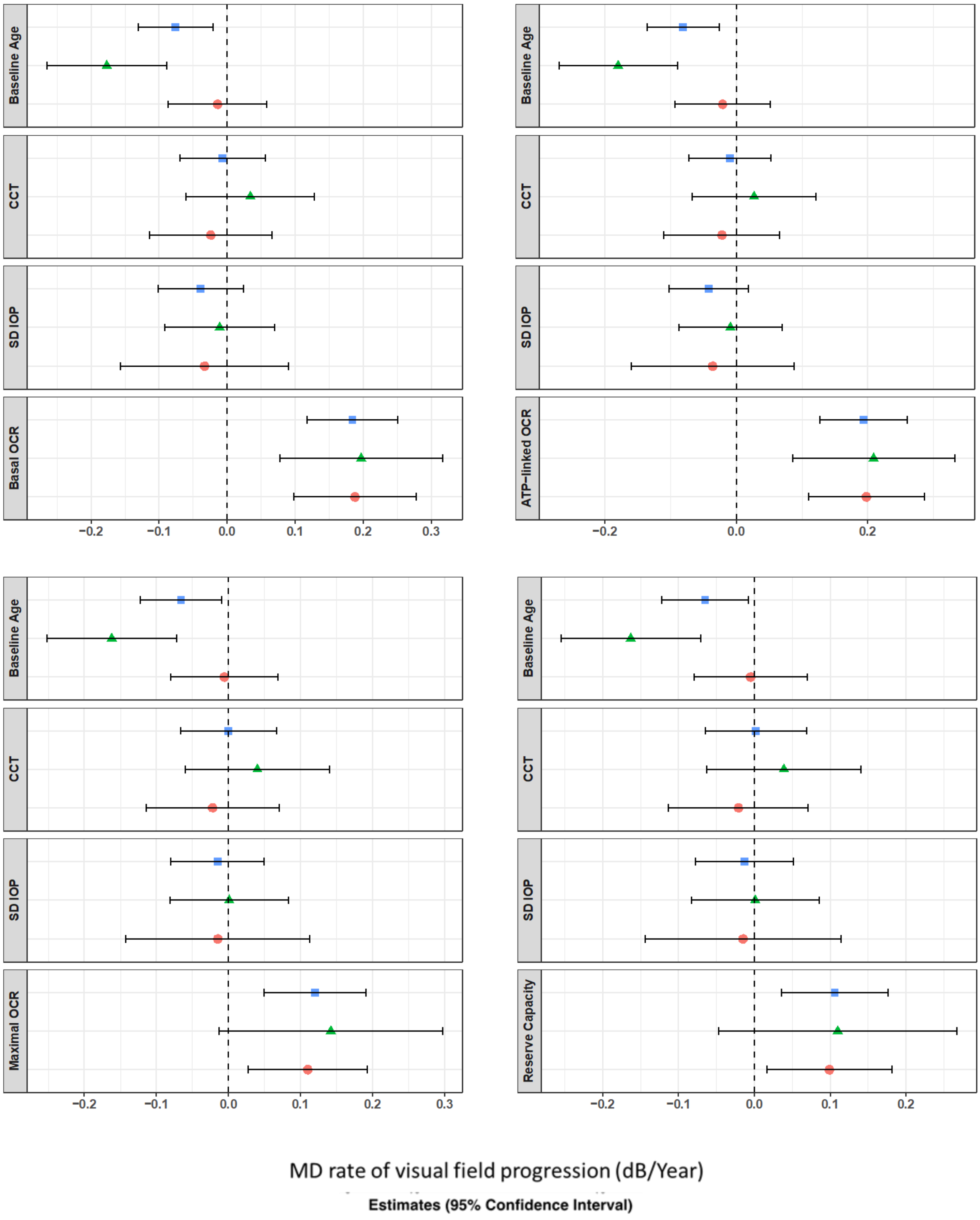

Supplementary Figure 11

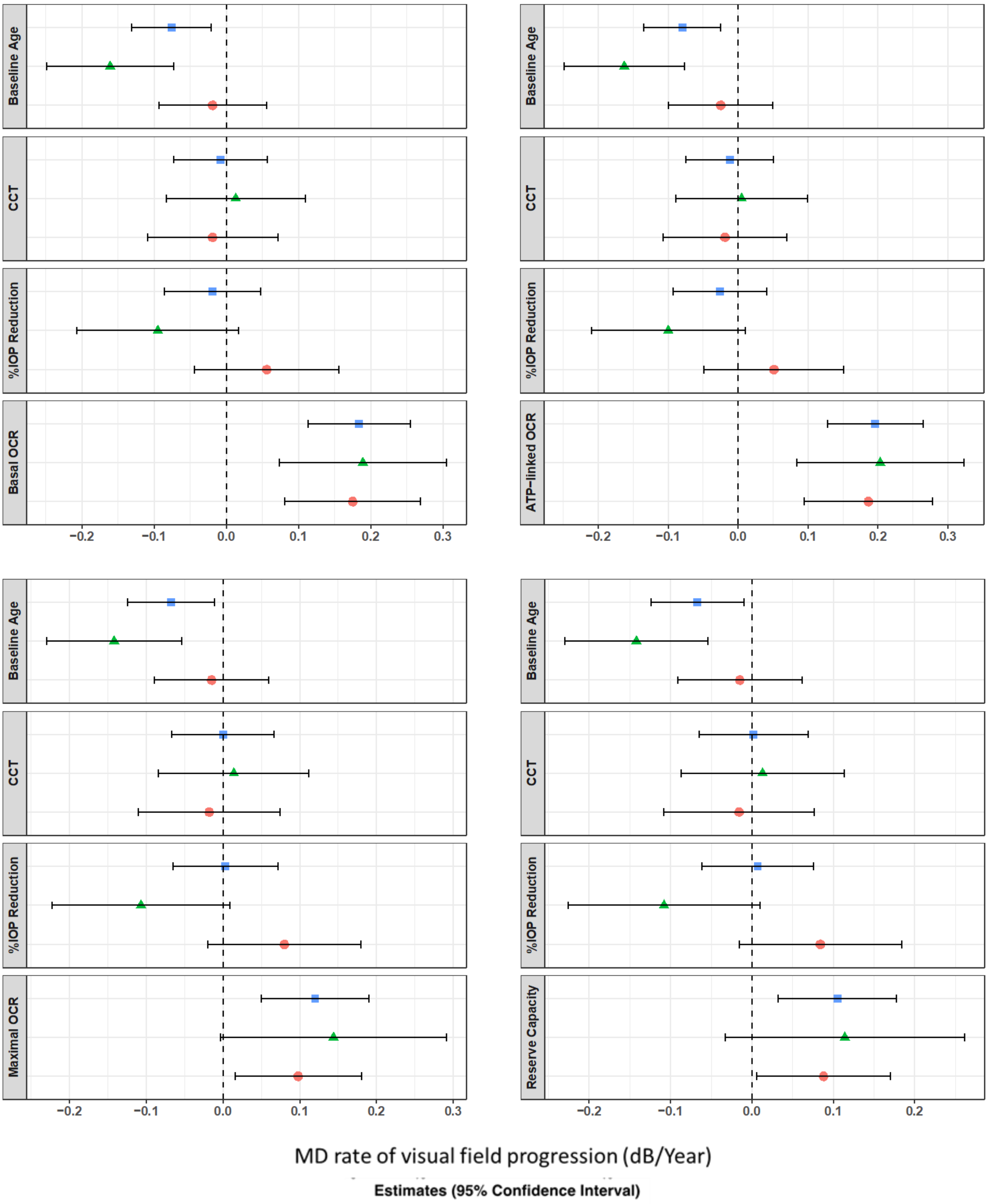

Sup. Fig. 8-11 Forest Plots showing the results of the various OCR mixed effect models for factors associated with the mean deviation (MD) visual field progression, expressed as dB/year. A total of 229 eyes (NTG: 144 eyes, HTG: 85 eyes) of 139 patients were included in this analysis. Blue squares, green triangles and red circles represent standardized Estimates, whereas horizontal bars represent their

corresponding 95% confidence intervals. All independent variable were standardized (i.e. zero mean and unit standard deviation). Each figure includes a different IOP metric.

Supplementary Figure 12

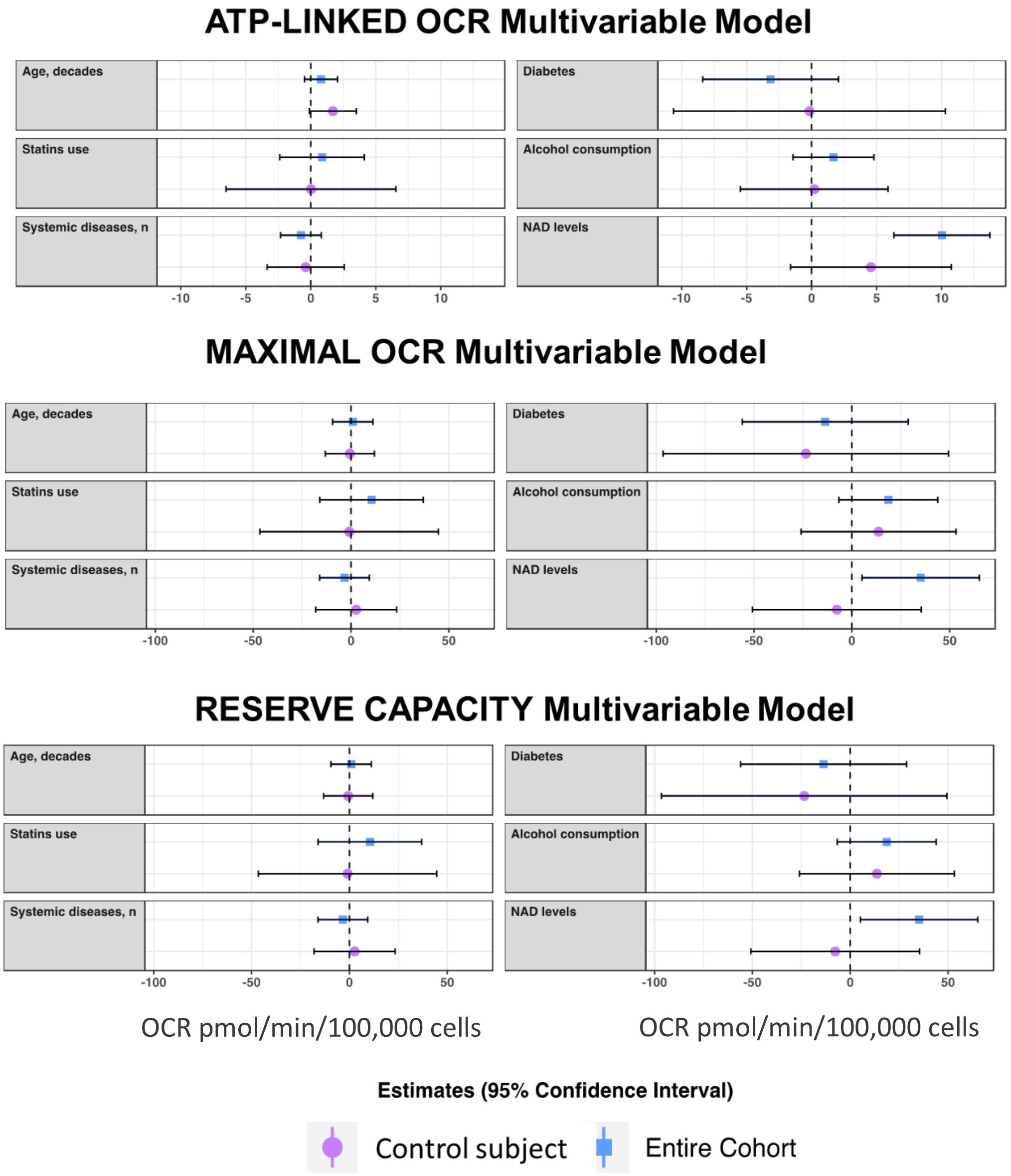

Sup. Fig. 12. Forest Plots showing the results of the multivariable model for factors associated with total cellular NAD levels and various OCR parameters (25 controls, 10 HTG and 19 NTG). Blue squares and purple circles represent beta estimates from the multivariable regression model, whereas horizontal bars represent their corresponding 95% confidence intervals.

Supplementary Figure 13

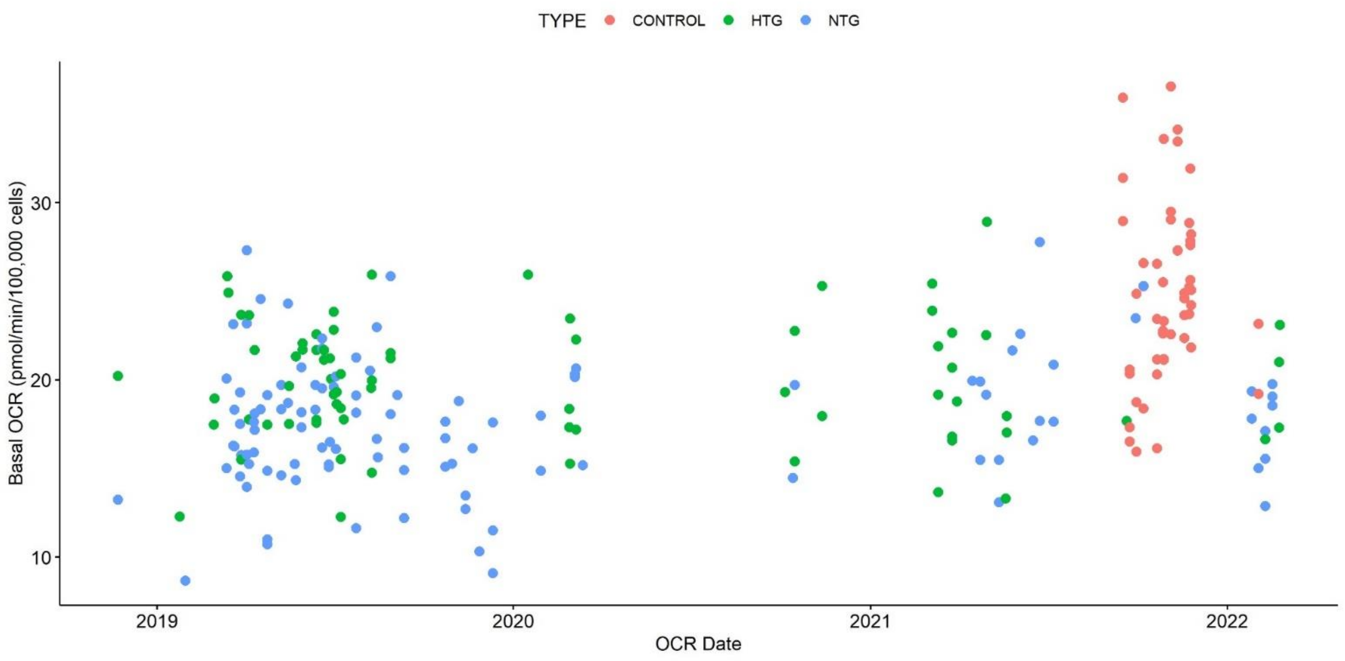

Sup. Fig. 13. Basal OCR over the course of the Seahorse assay dates for participants in the three groups. The HTG group is represented in green, NTG in blue, and Control in red.

Supplementary Figure 14

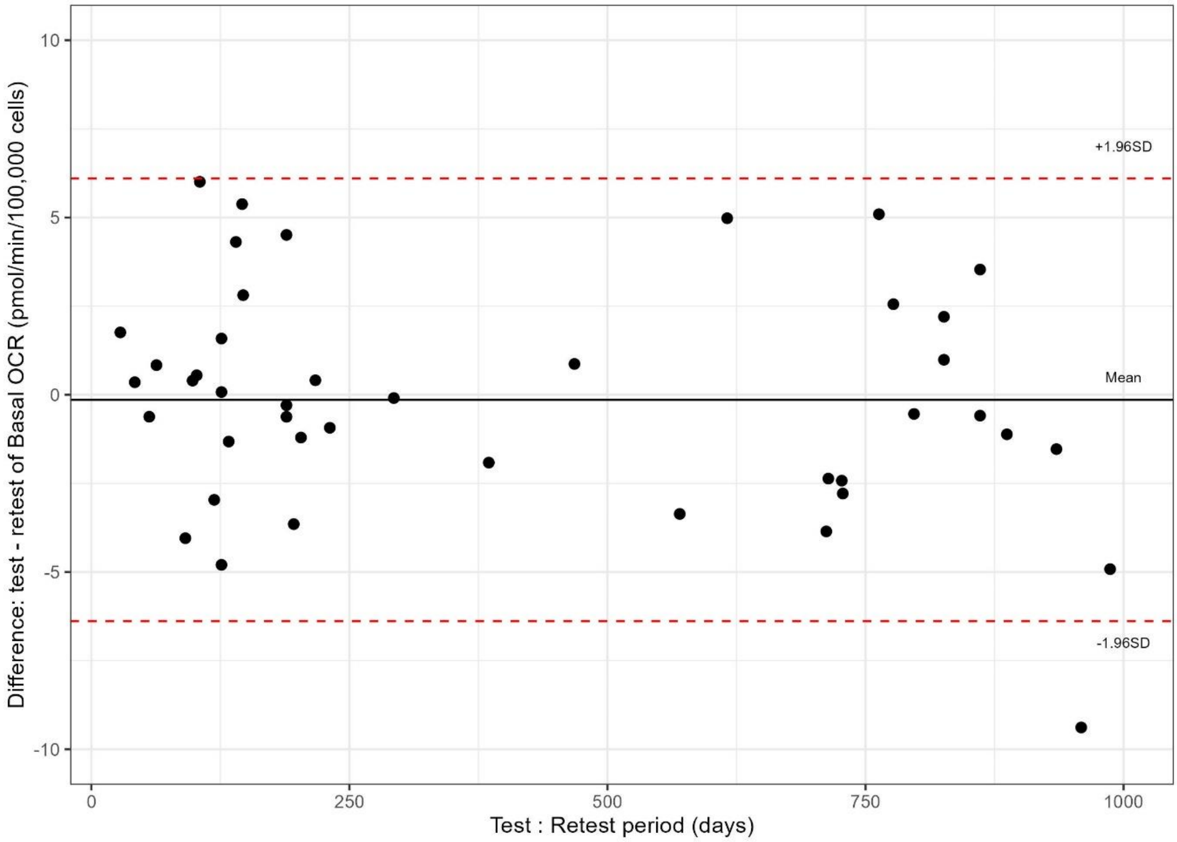

Sup.Fig.14. Bland-Altman plot for Basal OCR, with the y-axis representing the test-retest difference and the x-axis indicating the test-retest window in days. Results show a minimal bias of -0.1 pmol/min/100,000 cells. The limits of agreement (Upper limit of agreement: 6.1, Lower limit of agreement: -6.4) suggest that the variations observed are within an acceptable range, indicating consistent OCR measurements over time.

Supplementary Figure 15

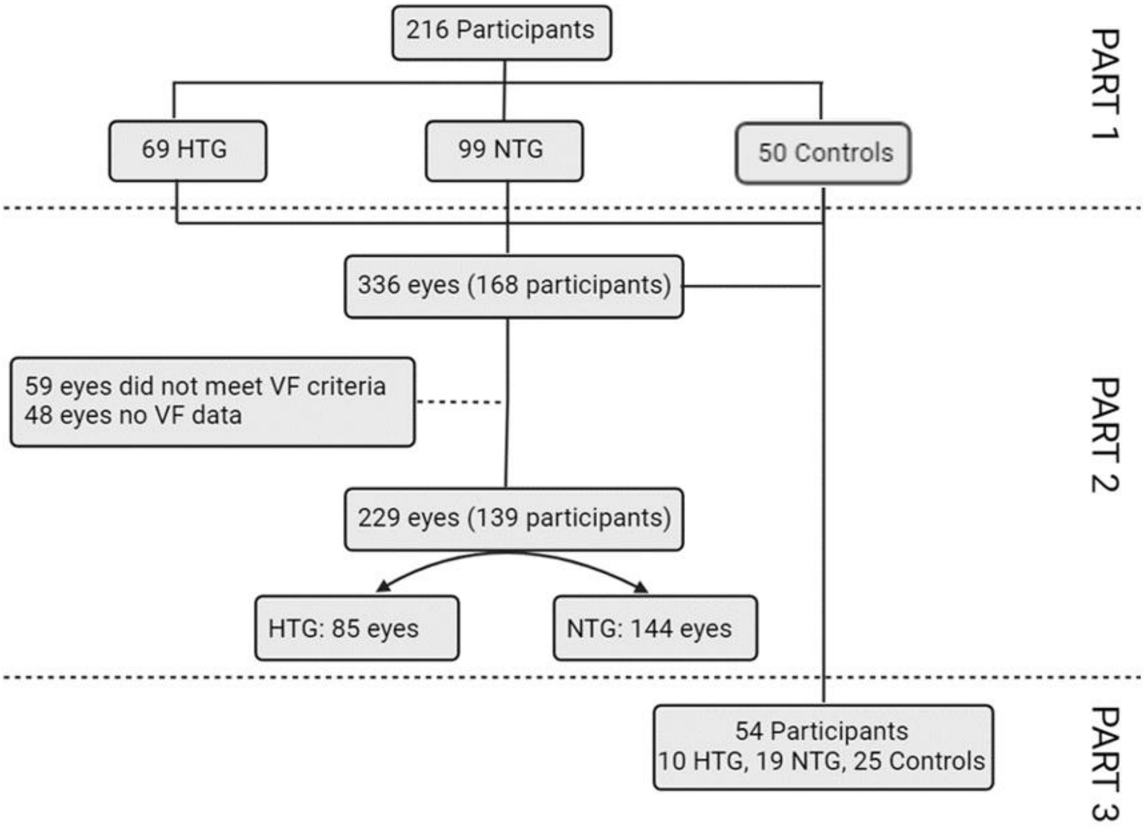

Sup. Fig. 15. Flow chart showing the number of participants/eyes included in each part of the study. Part 1 – measurement of oxygen consumption rate in PBMC. Part 2 – association of MD rate of visual field progression and oxygen consumption rate. Part 3 – measurement of total cellular NAD levels in PBMC.

Supplementary Figure 16

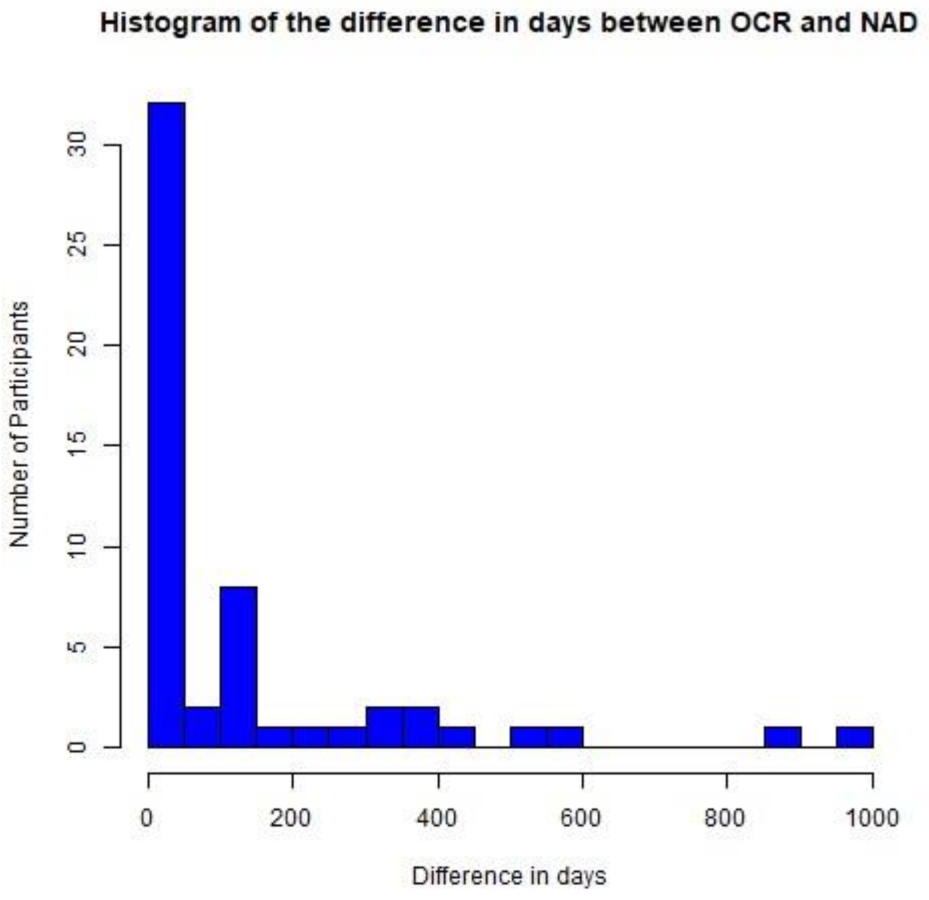

Sup. Fig. 16 Histogram showing the difference between OCR and NAD assays. Thirty-two out of fifty-four participants had both assays on the same day. Media (IQR) number of days between OCR and NAD assays was 0 (0 - 123) days.

Supplementary Figure 17

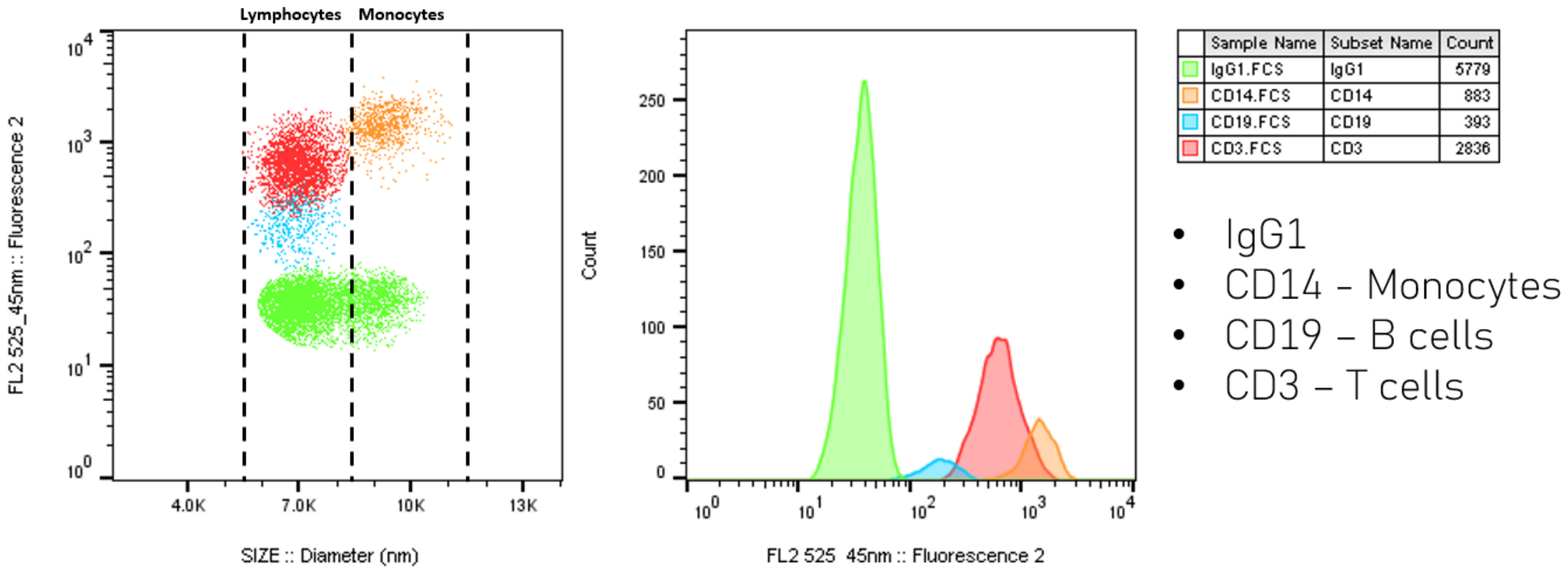

Supp. Fig. 17 Flow cytometry was performed on PBMC using antibodies for monocytes (CD14) and lymphocytes (CD3 – T cells, CD19 - B cells) as a positive control and IgG1 as a negative control. B and T cells (red and blue populations) lie between 5.5 – 8.5 µm, whereas monocytes (orange) above 8.5 µm.

**Supplementary Table 1.** Multivariable linear model for factors associated with basal OCR in the entire cohort, NTG, HTG and Healthy Controls								
	**Entire cohort**		**NTG**		**HTG**		**Healthy controls**	
Variable	β Estimate (SE)	P-value	β Estimate (SE)	P-value	β Estimate (SE)	P-value	β Estimate (SE)	P-value
Diagnostic status (ref: healthy subjects)								
HTG	-5.597 (0.834)	**<0.001**						
NTG	-8.514 (0.807)	**<0.001**						
Age, decades	0.890 (0.318)	**0.006**	0.489 (0.446)	0.276	0.122 (0.056)	**0.034**	0.105 (0.089)	0.244
Male sex	-0.532 (0.609)	0.384	-0.035 (0.842)	0.967	-1.671 (1.071)	0.124	0.323 (1.929)	0.868
Number of systemic diseases	-0.402 (0.312)	0.198	-0.495 (0.411)	0.232	-0.368 (0.591)	0.536	0.834 (0.961)	0.39
Diabetes	-1.376 (1.278)	0.283	0.004 (2.735)	0.999	-1.788 (1.776)	0.318	-1.82 (4.105)	0.66
Migraine	1.329 (0.841)	0.116	1.066 (1.112)	0.34	1.297 (1.333)	0.335	0.233 (3.290)	0.944
Vasospasm	1.195 (0.790)	0.132	1.394 (0.890)	0.121	0.662 (1.64)	0.688	2.256 (3.582)	0.533
Alcohol consumption	0.925 (0.706)	0.192	1.984 (0.987)	0.048	0.736 (1.203)	0.543	0.000 (2.524)	1

Systemic betablocker	-0.948 (0.850)	0.266	-2.216 (1.311)	0.094	0.883 (1.423)	0.537	-4.284 (3.135)	0.179	
BMI	-0.006 (0.065)	0.929	-0.087 (0.116)	0.457	-0.111 (0.101)	0.276	0.122 (0.156)	0.441	

Multivariable linear regression model for factors associated with Basal OCR. P-values less than 0.05 are indicated in bold text

Estimates for continuous variables are intended for 1-unit increase unless specified otherwise. Diagnostic status reference: control subject.

HTG: high-tension glaucoma; NTG: normal-tension glaucoma; OCR: oxygen consumption rate: SE: standard error.

**Supplementary Table 2.** Multivariable linear model for factors associated with ATP-linked OCR in the entire cohort, NTG, HTG and Healthy Controls								
	**Entire cohort**		**NTG**		**HTG**		**Healthy controls**	
Variable	β Estimate (SE)	P-value	β Estimate (SE)	P-value	β Estimate (SE)	P-value	β Estimate (SE)	P-value
Diagnostic status (ref: healthy subjects)								
HTG	-3.789 (0.702)	**<0.001**						
NTG	-6.235 (0.669)	**<0.001**						
Age, decades	0.878 (0.266)	**0.001**	0.609 (0.367)	0.101	0.829 (0.443)	0.066	1.254 (0.756)	0.105
Male sex	-0.733 (0.517)	0.158	0.221 (0.703)	0.754	-1.12 (0.873)	0.204	-1.098 (1.633)	0.505
Number of systemic diseases	-0.577 (0.291)	**0.049**	-0.912 (0.357)	**0.012**	-0.066 (0.551)	0.904	-0.493 (0.914)	0.593
Diabetes	-0.701 (1.088)	0.52	0.711 (2.15)	0.742	-1.403 (1.403)	0.321	-1.657 (3.586)	0.647
Alcohol consumption	0.672 (0.601)	0.264	1.552 (0.797)	0.055	0.525 (0.984)	0.596	0.747 (2.062)	0.719
Statin use	0.459 (0.642)	0.475	-0.912 (0.357)	**0.012**	-1.291 (1.043)	0.22	-0.493 (0.914)	0.593
BMI	-0.019 (0.055)	0.736	-0.078 (0.095)	0.415	-0.069 (0.08)	0.391	0.067 (0.136)	0.628

Multivariable linear regression model for factors associated with ATP-linked OCR. P-values less than 0.05 are indicated in bold text Estimates for continuous variables are intended for 1-unit increase unless specified otherwise. Diagnostic status reference: control subject.

ATP: adenosine triphosphate; HTG: high-tension glaucoma; NTG: normal-tension glaucoma; OCR: oxygen consumption rate: SE: standard error.

**Supplementary Table 3.** Multivariable linear model for factors associated with Maximal OCR in the entire cohort, NTG, HTG and Healthy Controls								
	**Entire cohort**		**NTG**		**HTG**		**Healthy controls**	
Variable	β Estimate (SE)	P-value	β Estimate (SE)	P-value	β Estimate (SE)	P-value	β Estimate (SE)	P-value
Diagnostic status (ref: healthy subjects)								
HTG	-22.812 (6.214)	**<0.001**						
NTG	-36.715 (5.922)	**<0.001**						
Age, decades	2.298 (2.357)	0.331	3.311 (3.906)	0.399	-1.753 (3.805)	0.647	1.36 (4.701)	0.774
Number of systemic diseases	-7.914 (2.58)	**0.002**	-13.286 (3.799)	**0.001**	-2.81 (4.903)	0.569	0.291 (5.512)	0.958
Diabetes	-11.114 (9.589)	0.248	-4.018 (22.88)	0.861	-5.916 (12.513)	0.638	-52.026 (20.838)	**0.016**
Alcohol consumption	10.458 (5.202)	**0.046**	17.921 (8.483)	**0.037**	9.903 (8.016)	0.221	6.392 (12.387)	0.609
Statin use	13.737 (5.681)	**0.016**	25.868 (9.1)	**0.006**	-1.173 (9.126)	0.898	4.978 (13.142)	0.707
BMI	-0.233 (0.49)	0.635	-0.9 (1.001)	0.371	-0.649 (0.711)	0.365	1.259 (0.848)	0.145

Multivariable linear regression model for factors associated with Maximal OCR. P-values less than 0.05 are indicated in bold text

Estimates for continuous variables are intended for 1-unit increase unless specified otherwise. Diagnostic status reference: control subject.

HTG: high-tension glaucoma; NTG: normal-tension glaucoma; OCR: oxygen consumption rate: SE: standard error.

Supplementary Table 4. Multivariable linear model for factors associated with Reserve OCR in the entire cohort, NTG, HTG and Healthy Controls

	**Entire cohort**		**NTG**		**HTG**		**Healthy controls**	
Variable	β Estimate (SE)	P-value	β Estimate (SE)	P-value	β Estimate (SE)	P-value	β Estimate (SE)	P-value
Diagnostic status (ref: healthy subjects)								
HTG	-17.483 (5.754)	**0.003**						
NTG	-28.72 (5.484)	**<0.001**						
Age, decades	1.478 (2.183)	0.499	2.891 (3.628)	0.428	-2.514 (3.535)	0.48	0.146 (4.317)	0.973
Number of systemic diseases	-7.249 (2.389)	**0.003**	-12.064 (3.529)	**0.001**	-3.259 (4.556)	0.477	0.877 (5.062)	0.863
Diabetes	-9.808 (8.879)	0.271	-4.146 (21.25)	0.846	-3.445 (11.625)	0.768	-48.791 (19.136)	**0.014**
Alcohol consumption	9.895 (4.817)	**0.041**	16.279 (7.879)	**0.042**	9.582 (7.447)	0.203	5.406 (11.375)	0.637
Statin use	13.085 (5.261)	**0.014**	23.486 (8.452)	**0.007**	1.041 (8.479)	0.903	3.181 (12.068)	0.793
BMI	-0.208 (0.454)	0.647	-0.799 (0.93)	0.392	-0.57 (0.66)	0.391	1.141 (0.779)	0.15

Multivariable linear regression model for factors associated with Reserve OCR. P-values less than 0.05 are indicated in bold text

Estimates for continuous variables are intended for 1-unit increase unless specified otherwise. Diagnostic status reference: control subject.

HTG: high-tension glaucoma; NTG: normal-tension glaucoma; OCR: oxygen consumption rate: SE: standard error

Supplementary Table 5. Standardized multivariable linear mixed model for factors associated with visual field mean deviation (MD) rates of change in entire cohort.

	**Model A**		**Model B**		**Model C**		**Model D**	
**Variable**	*β Estimate (SE)*	*P-value*	*β Estimate (SE)*	*P-value*	*β Estimate (SE)*	*P-value*	*β Estimate (SE)*	*P-value*
Age	-0.090 (0.029)	**0.002**	-0.097 (0.029)	**0.001**	-0.076 (0.029)	**0.010**	-0.075 (0.030)	**0.012**
CCT	0.008 (0.033)	0.82	0.005 (0.029)	0.87	0.012 (0.034)	0.73	0.013 (0.035)	0.70
Mean IOP	-0.069 (0.034)	**0.042**	-0.076 (0.033)	**0.025**	-0.057 (0.035)	0.10	-0.056 (0.035)	0.11
Basal OCR	0.186 (0.035)	**<0.001**						
ATP-linked OCR			0.199 (0.034)	**<0.001**				
Maximal OCR					0.123 (0.036)	**0.001**		
Reserve Capacity							0.108 (0.036)	**0.003**

The relationship between the rate of MD change and OCR was evaluated with linear mixed models with random slopes and random intercepts. P-values less than 0.05 are indicated in bold text

The current model has mean intraocular pressure (IOP) as IOP covariate. All covariates are standardized (i.e., zero mean and unit standard deviation), and coefficient effect should be interpreted as one standard deviation increase in the dependent variable.

ATP: adenosine triphosphate; CCT: central corneal thickness; OCR: oxygen consumption rate.

Supplementary Table 6. Standardized multivariable linear mixed model for factors associated with visual field mean deviation (MD) rates of change in the normal-tension glaucoma (NTG) cohort.

	**Model A**		**Model B**		**Model C**		**Model D**	
**Variable**	*β Estimate (SE)*	*P-value*	*β Estimate (SE)*	*P-value*	*β Estimate (SE)*	*P-value*	*β Estimate (SE)*	*P-value*
Age	-0.034 (0.040)	0.40	-0.040 (0.039)	0.31	-0.032 (0.040)	0.43	-0.031 (0.041)	0.44
CCT	-0.026 (0.046)	0.57	-0.025 (0.045)	0.59	-0.025 (0.047)	0.60	-0.024 (0.047)	0.62
Mean IOP	-0.108 (0.067)	0.11	-0.109 (0.066)	0.11	-0.133 (0.069)	0.056	-0.137 (0.069)	0.050
Basal OCR	0.176 (0.047)	**<0.001**						
ATP-linked OCR			0.187 (0.046)	**<0.001**				
Maximal OCR					0.102 (0.042)	**0.018**		
Reserve Capacity							0.092 (0.042)	**0.033**

The relationship between the rate of MD change and OCR was evaluated with linear mixed models with random slopes and random intercepts. P-values less than 0.05 are indicated in bold text

The current model has mean intraocular pressure (IOP) as IOP covariate. All variables are standardized (i.e., zero mean and unit standard deviation), and coefficient effect should be interpreted as one standard deviation increase in the dependent variable.

ATP: adenosine triphosphate; CCT: central corneal thickness; OCR: oxygen consumption rate.

Supplementary Table 7. Standardized multivariable linear mixed model for factors associated with visual field mean deviation (MD) rates of change in the high-tension glaucoma (HTG) cohort.

	**Model A**		**Model B**		**Model C**		**Model D**	
**Variable**	*β Estimate (SE)*	*P-value*	*β Estimate (SE)*	*P-value*	*β Estimate (SE)*	*P-value*	*β Estimate (SE)*	*P-value*
Age	-0.195 (0.047)	**<0.001**	-0.198 (0.047)	**<0.001**	-0.179 (0.047)	**<0.001**	-0.179 (0.048)	**<0.001**
CCT	0.062 (0.052)	0.25	0.057 (0.052)	0.28	0.071 (0.055)	0.20	0.070 (0.055)	0.21
Mean IOP	-0.083 (0.058)	0.16	-0.088 (0.057)	0.13	-0.093 (0.060)	0.12	-0.095 (0.061)	0.12
Basal OCR	0.197 (0.061)	**0.002**						
ATP-linked OCR			0.211 (0.063)	**0.002**				
Maximal OCR					0.142 (0.079)	0.08		
Reserve Capacity							0.110 (0.079)	0.17

The relationship between the rate of MD change and OCR was evaluated with linear mixed models with random slopes and random intercepts. P-values less than 0.05 are indicated in bold text

The current model has mean intraocular pressure (IOP) as IOP covariate. All variables are standardized (i.e., zero mean and unit standard deviation), and coefficient effect should be interpreted as one standard deviation increase in the dependent variable.

ATP: adenosine triphosphate; CCT: central corneal thickness; OCR: oxygen consumption rate.

Supplementary Table 8. Standardized multivariable linear mixed model for factors associated with visual field mean deviation (MD) rates of change in the entire cohort.

	**Model A**		**Model B**		**Model C**		**Model D**	
**Variable**	*β Estimate (SE)*	*P-value*	*β Estimate (SE)*	*P-value*	*β Estimate (SE)*	*P-value*	*β Estimate (SE)*	*P-value*
Age	-0.090 (0.029)	**0.002**	-0.097 (0.029)	**0.001**	-0.078 (0.029)	**0.008**	-0.076 (0.030)	**0.010**
CCT	0.008 (0.033)	0.82	0.005 (0.033)	0.87	0.013 (0.034)	0.71	0.014 (0.035)	0.68
Mean IOP	-0.069 (0.035)	**0.047**	-0.076 (0.034)	**0.030**	-0.059 (0.035)	0.09	-0.057 (0.035)	0.10
Basal OCR	0.186 (0.035)	**<0.001**						
ATP-linked OCR			0.199 (0.034)	**<0.001**				
Maximal OCR					0.126 (0.036)	**<0.001**		
Reserve Capacity							0.111 (0.036)	**0.003**
Mean IOP*OCR parameter	0.001 (0.031)	0.98	0.000 (0.028)	0.99	0.031 (0.037)	0.41	0.031 (0.038)	0.41

The relationship between the rate of MD change and OCR was evaluated with linear mixed models with random slopes and random intercepts. P-values less than 0.05 are indicated in bold text

The current model has mean intraocular pressure (IOP) as IOP covariate. All variables are standardized (i.e., zero mean and unit standard deviation), and coefficient effect should be interpreted as one standard deviation increase in the dependent variable.

ATP: adenosine triphosphate; CCT: central corneal thickness; OCR: oxygen consumption rate.

**Supplementary Table 9.** Standardized multivariable linear mixed model for factors associated with visual field mean deviation (MD) rates of change in the entire cohort.								
	**Model A**		**Model B**		**Model C**		**Model D**	
**Variable**	*ß Estimate (SE)*	*P-value*	*ß Estimate (SE)*	*P-value*	*ß Estimate (SE)*	*P-value*	*ß Estimate (SE)*	*P-value*
Age	-0.084 (0.028)	**0.003**	-0.090 (0.028)	**0.001**	-0.071 (0.029)	**0.015**	-0.069 (0.029)	**0.018**
CCT	0.003 (0.032)	0.93	0.000 (0.032)	0.99	0.007 (0.034)	0.84	0.008 (0.034)	0.81
Peak IOP	-0.075 (0.033)	**0.024**	-0.079 (0.033)	**0.016**	-0.051 (0.034)	0.14	-0.048 (0.034)	0.16
Basal OCR	0.190 (0.034)	**<0.001**						
ATP-linked OCR			0.202 (0.034)	**<0.001**				
Maximal OCR					0.122 (0.035)	**0.001**		
Reserve Capacity							0.107 (0.036)	**0.003**

The relationship between the rate of MD change and OCR was evaluated with linear mixed models with random slopes and random intercepts. P-values less than 0.05 are indicated in bold text

The current model has peak intraocular pressure (IOP) as IOP covariate. All variables are standardized (i.e., zero mean and unit standard deviation), and coefficient effect should be interpreted as one standard deviation increase in the dependent variable.

ATP: adenosine triphosphate; CCT: central corneal thickness; OCR: oxygen consumption rate.

Supplementary Table 10. Standardized multivariable linear mixed model for factors associated with visual field mean deviation (MD) rates of change in the entire cohort.

	**Model A**		**Model B**		**Model C**		**Model D**	
**Variable**	*β Estimate (SE)*	*P-value*	*β Estimate (SE)*	*P-value*	*β Estimate (SE)*	*P-value*	*β Estimate (SE)*	*P-value*
Age	-0.076 (0.028)	**0.007**	-0.081 (0.028)	**0.004**	-0.066 (0.029)	**0.022**	-0.065 (0.029)	**0.025**
CCT	-0.007 (0.032)	0.84	-0.010 (0.032)	0.75	0.000 (0.034)	0.99	0.002 (0.034)	0.96
IOP Fluctuation	-0.039 (0.032)	0.22	-0.042 (0.031)	0.19	-0.015 (0.033)	0.65	-0.013 (0.033)	0.70
Basal OCR	0.184 (0.034)	**<0.001**						
ATP-linked OCR			0.194 (0.034)	**<0.001**				
Maximal OCR					0.120 (0.036)	**0.001**		
Reserve Capacity							0.106 (0.036)	**0.004**

The relationship between the rate of MD change and OCR was evaluated with linear mixed models with random slopes and random intercepts. P-values less than 0.05 are indicated in bold text

The current model has intraocular pressure (IOP) fluctuation as IOP covariate. This is SD of IOPs over the visits in the study period. All variables are standardized (i.e., zero mean and unit standard deviation), and coefficient effect should be interpreted as one standard deviation increase in the dependent variable.

ATP: adenosine triphosphate; CCT: central corneal thickness; OCR: oxygen consumption rate.

Supplementary Table 11. Standardized multivariable linear mixed model for factors associated with visual field mean deviation (MD) rates of change in the entire cohort.

	**Model A**		**Model B**		**Model C**		**Model D**	
**Variable**	*β Estimate (SE)*	*P-value*	*β Estimate (SE)*	*P-value*	*β Estimate (SE)*	*P-value*	*β Estimate (SE)*	*P-value*
Age	-0.076 (0.028)	**0.008**	-0.080 (0.028)	**0.005**	-0.068 (0.029)	**0.021**	-0.067 (0.029)	**0.023**
CCT	-0.008 (0.033)	0.81	-0.012 (0.032)	0.71	0.000 (0.034)	0.99	0.002 (0.034)	0.94
Relative IOP reduction	-0.019 (0.034)	0.58	-0.026 (0.034)	0.44	0.003 (0.035)	0.94	0.007 (0.035)	0.84
Basal OCR	0.184 (0.036)	**<0.001**						
ATP-linked OCR			0.196 (0.035)	**<0.001**				
Maximal OCR					0.120 (0.036)	**0.001**		
Reserve Capacity							0.105 (0.037)	**0.005**

The relationship between the rate of MD change and OCR was evaluated with linear mixed models with random slopes and random intercepts. P-values less than 0.05 are indicated in bold text

The current model has relative intraocular pressure (IOP) reduction from baseline as IOP covariate. All variables are standardized (i.e., zero mean and unit standard deviation), and coefficient effect should be interpreted as one standard deviation increase in the independent variable.

ATP: adenosine triphosphate; CCT: central corneal thickness; OCR: oxygen consumption rate.

**Supplementary Table 12.** Standardized multivariable linear mixed model for factors associated with visual field mean deviation (MD) rates of change in subset of patients undergoing surgery.								
	**Model A**		**Model B**		**Model C**		**Model D**	
**Variable**	*β Estimate (SE)*	*P-value*	*β Estimate (SE)*	*P-value*	*β Estimate (SE)*	*P-value*	*β Estimate (SE)*	*P-value*
Age, per decade	-0.160 (0.040)	**<0.001**	-0.166 (0.040)	**<0.001**	-0.158 (0.040)	**<0.001**	-0.157 (0.040)	**<0.001**
CCT, per 10 µm	-0.007 (0.050)	0.90	-0.011 (0.050)	0.82	-0.022 (0.051)	0.67	-0.022 (0.051)	0.67
Mean IOP	-0.048 (0.051)	0.35	-0.047 (0.051)	0.36	-0.052 (0.052)	0.32	-0.053 (0.052)	0.31
Basal OCR	0.115 (0.046)	**<0.001**						
ATP-linked OCR			0.147 (0.047)	**0.003**				
Maximal OCR					0.073 (0.046)	0.12		
Reserve Capacity							0.064 (0.046)	0.17

The relationship between the rate of MD change and OCR was evaluated with linear mixed models with random slopes and random intercepts. P-values less than 0.05 are indicated in bold text

The current model has mean intraocular pressure (IOP) as IOP covariate. All covariates are standardized (i.e., zero mean and unit standard deviation), and coefficient effect should be interpreted as one standard deviation increase in the dependent variable.

ATP: adenosine triphosphate; CCT: central corneal thickness; OCR: oxygen consumption rate.

Supplementary Table 13. Standardized multivariable linear mixed model for factors associated with visual field mean deviation (MD) rates of change in subset of patients not undergoing surgery.

	**Model A**		**Model B**		**Model C**		**Model D**	
**Variable**	*β Estimate (SE)*	*P-value*	*β Estimate (SE)*	*P-value*	*β Estimate (SE)*	*P-value*	*β Estimate (SE)*	*P-value*
Age, per decade	-0.118 (0.041)	**0.005**	-0.015 (0.042)	**0.007**	-0.080 (0.042)	0.06	-0.075 (0.042)	0.08
CCT, per 10 µm	-0.046 (0.039)	0.25	-0.033 (0.040)	0.42	-0.007 (0.041)	0.86	-0.002 (0.041)	0.96
Mean IOP	-0.118 (0.041)	**0.005**	-0.121 (0.042)	**0.005**	-0.067 (0.041)	0.11	-0.061 (0.042)	0.15
Basal OCR	0.282 (0.047)	**<0.001**						
ATP-linked OCR			0.258 (0.047)	**<0.001**				
Maximal OCR					0.207 (0.047)	**<0.001**		
Reserve Capacity							0.190 (0.048)	**<0.001**

The relationship between the rate of MD change and OCR was evaluated with linear mixed models with random slopes and random intercepts. P-values less than 0.05 are indicated in bold text

The current model has mean intraocular pressure (IOP) as IOP covariate. All covariates are standardized (i.e., zero mean and unit standard deviation), and coefficient effect should be interpreted as one standard deviation increase in the dependent variable.

ATP: adenosine triphosphate; CCT: central corneal thickness; OCR: oxygen consumption rate.

Supplementary Table 14. Standardized multivariable linear mixed model for factors associated with visual field mean deviation (MD) rates of change in entire cohort.

	Model A		Model B		Model C		Model D	
**Variable**	*ß Estimate (SE)*	*P-value*	*ß Estimate (SE)*	*P-value*	*ß Estimate (SE)*	*P-value*	*ß Estimate (SE)*	*P-value*
Age, per decade	-0.135 (0.028)	**<0.001**	-0.138 (0.029)	**<0.001**	-0.119 (0.019)	**<0.001**	-0.117 (0.029)	**<0.001**
CCT, per 10 μm	-0.027 (0.031)	0.39	-0.023 (0.031)	0.47	-0.015 (0.032)	0.64	-0.013 (0.032)	0.69
Mean IOP	-0.079 (0.032)	**0.013**	-0.081 (0.032)	**0.012**	-0.056 (0.032)	0.08	-0.053 (0.032)	0.10
Glaucoma surgery (ref:no)	-0.401 (0.064)	**<0.001**	-0.393 (0.064)	**<0.001**	-0.426 (0.065)	**<0.001**	-0.428 (0.066)	**<0.001**
OCR*Surgery	-0.167 (0.063)	**0.009**	-0.114 (0.063)	0.07	-0.144 (0.064)	**0.026**	-0.135 (0.064)	**0.037**
Basal OCR	0.270 (0.047)	**<0.001**						
ATP-linked OCR			0.248 (0.045)	**<0.001**				
Maximal OCR					0.211 (0.047)	**<0.001**		
Reserve Capacity							0.193 (0.048)	**<0.001**

The relationship between the rate of MD change and OCR was evaluated with linear mixed models with random slopes and random intercepts. P-values less than 0.05 are indicated in bold text

The current model has mean intraocular pressure (IOP) as IOP covariate. All covariates are standardized (i.e., zero mean and unit standard deviation), and coefficient effect should be interpreted as one standard deviation increase in the dependent variable.

ATP: adenosine triphosphate; CCT: central corneal thickness; OCR: oxygen consumption rate.

**Supplementary Table 15.** Demographic and clinical characteristics of the placebo group included in the UKGTS trial	
**Variable**	**Entire cohort**
Number of eyes/patients	213/213
Baseline Age, mean (±SD)	66.5 (±10.3)
Sex (female/male)	108/105
Laterality, right/left	92/121
CCT, mean (±SD), micron	544 (±34)
Baseline MD, median (IQR), dB	-3.4 (-2.0 to -5.6)
Baseline IOP, median (IQR), mmHg	19.5 (16.0 to 22.8)
Number of VF, median (IQR)	13 (10 to 16)
Follow-up time, median (IQR), years	1.6 (1.0 to 2.0)
CCT: central corneal thickness; dB: decibel; IOP: intraocular pressure; IQR: interquartile range; MD: mean deviation; SD: standard deviation; UKGTS: United Kingdom Glaucoma Treatment Study; VF: visual field.	

**Supplementary Table 16.** Multivariable linear model for factors associated with basal OCR in the entire cohort and non-glaucomatous controls								
	**Entire cohort**		**Controls**		**NTG**		**HTG**	
**Variable**	*ß Estimate (SE)*	*P-value*	*ß Estimate (SE)*	*P-value*	*ß Estimate (SE)*	*P-value*	*ß Estimate (SE)*	*P-value*
Age, decades	0.400 (0.730)	0.59	1.397 (0.980)	0.17	-0.954 (1.211)	0.44	-4.368 (9.927)	0.69
Statins use	1.585 (1.871)	0.40	2.029 (3.549)	0.58	1.101 (3.225)	0.74	-2.820 (6.344)	0.69
Number of systemic diseases	-0.817 (0.898)	0.37	-1.132 (1.612)	0.49	-1.428 (1.370)	0.32	0.586 (3.536)	0.89
Diabetes	-4.262 (2.998)	0.16	-1.962 (5.678)	0.74	(*)	(*)	-6.505 (16.724)	0.72
Alcohol consumption	2.363 (1.786)	0.19	-0.263 (3.081)	0.93	6.950 (2.76)	**0.026**	-5.704 (14.763)	0.72
NAD levels	11.601 (2.120)	**<0.001**	6.467 (3.358)	0.07	1.803 (7.562)	0.82	11.720 (20.971)	0.62

Multivariable linear regression model for factors associated with basal OCR. P-values less than 0.05 are indicated in bold text

Estimates for continuous variables are intended for 1-unit increase unless specified otherwise.

HTG: high-tension glaucoma; NTG: normal-tension glaucoma; OCR: oxygen consumption rate: SE: standard error.

(*) All NTG participants had Diabetes Type II, therefore diabetes could not be included as a variable in the model.

**Supplementary Table 17.** Multivariable linear model for factors associated with ATP-linked OCR in the entire cohort and non-glaucomatous controls				
	**Entire cohort**		**Controls**	
**Variable**	*β Estimate (SE)*	*P-value*	*β Estimate (SE)*	*P-value*
Age, decades	0.788 (0.648)	0.23	1.698 (0.920)	0.08
Statins use	0.870 (1.660)	0.60	0.012 (3.333)	0.99
Number of systemic diseases	-0.756 (0.797)	0.35	-0.390 (1.514)	0.80
Diabetes	-3.158 (2.661)	0.24	-0.162 (5.333)	0.98
Alcohol consumption	1.681 (1.585)	0.29	0.207 (2.894)	0.94
NAD levels	10.024 (1.882)	**<0.001**	4.561 (3.154)	0.17

Multivariable linear regression model for factors associated with ATP-linked OCR. P-values less than 0.05 are indicated in bold text

Estimates for continuous variables are intended for 1-unit increase unless specified otherwise.

HTG: high-tension glaucoma; NTG: normal-tension glaucoma; OCR: oxygen consumption rate: SE: standard error.

**Supplementary Table 18.** Multivariable linear model for factors associated with maximal OCR in the entire cohort and non-glaucomatous controls				
	**Entire cohort**		**Controls**	
**Variable**	*β Estimate (SE)*	*P-value*	*β Estimate (SE)*	*P-value*
Age, decades	0.895 (5.268)	0.87	-0.619 (6.421)	0.92
Statins use	10.500 (13.501)	0.44	-0.966 (23.260)	0.98
Number of systemic diseases	-3.318 (6.483)	0.61	2.644 (10.566)	0.81
Diabetes	-13.595 (21.636)	0.53	-23.525 (37.217)	0.54
Alcohol consumption	18.690 (12.888)	0.15	13.651 (20.195)	0.51
NAD levels	35.207 (15.302)	**0.026**	-7.692 (22.009)	0.73

Multivariable linear regression model for factors associated with Maximal OCR. P-values less than 0.05 are indicated in bold text

Estimates for continuous variables are intended for 1-unit increase unless specified otherwise.

HTG: high-tension glaucoma; NTG: normal-tension glaucoma; OCR: oxygen consumption rate: SE: standard error.

Supplementary Table 19. Multivariable linear model for factors associated with reserve OCR in the entire cohort and non-glaucomatous controls

	Entire cohort		Controls	
**Variable**	*β Estimate (SE)*	*P-value*	*β Estimate (SE)*	*P-value*
Age, decades	0.895 (5.268)	0.87	-0.619 (6.421)	0.92
Statins use	10.500 (13.501)	0.44	-0.966 (23.260)	0.97
Number of systemic diseases	-3.318 (6.483)	0.61	2.644 (10.566)	0.81
Diabetes	-13.595 (21.636)	0.53	-23.525 (37.217)	0.54
Alcohol consumption	18.690 (12.888)	0.15	13.651 (20.195)	0.51
NAD levels	35.207 (15.302)	**0.026**	-7.692 (22.009)	0.73

Multivariable linear regression model for factors associated with Reserve OCR. P-values less than 0.05 are indicated in bold text

Estimates for continuous variables are intended for 1-unit increase unless specified otherwise.

HTG: high-tension glaucoma; NTG: normal-tension glaucoma; OCR: oxygen consumption rate: SE: standard error.
