# Supplementary material for: Peripheral blood mononuclear cell respiratory function is associated with progressive glaucomatous vision loss
Source: Nat Med. 2024 Jun 17;30(8):2362–70. doi: 10.1038/s41591-024-03068-6 (PMC11333286; doi:10.1038/s41591-024-03068-6)
Supplement: Supplementary file 2 — Reporting Summary [file 41591_2024_3068_MOESM2_ESM.pdf]

nature portfolio

Corresponding author(s): David F Garway-Heath

Last updated by author(s): Apr 30, 2024

Reporting Summary

Nature Portfolio wishes to improve the reproducibility of the work that we publish. This form provides structure for consistency and transparency in reporting. For further information on Nature Portfolio policies, see our Editorial Policies and the Editorial Policy Checklist.

Statistics

For all statistical analyses, confirm that the following items are present in the figure legend, table legend, main text, or Methods section.

n/a	Confirmed	
☐	☒	The exact sample size (n) for each experimental group/condition, given as a discrete number and unit of measurement
☐	☒	A statement on whether measurements were taken from distinct samples or whether the same sample was measured repeatedly
☐	☒	The statistical test(s) used AND whether they are one- or two-sided<br>*Only common tests should be described solely by name; describe more complex techniques in the Methods section.*
☐	☒	A description of all covariates tested
☐	☒	A description of any assumptions or corrections, such as tests of normality and adjustment for multiple comparisons
☐	☒	A full description of the statistical parameters including central tendency (e.g. means) or other basic estimates (e.g. regression coefficient) AND variation (e.g. standard deviation) or associated estimates of uncertainty (e.g. confidence intervals)
☐	☒	For null hypothesis testing, the test statistic (e.g. F, t, r) with confidence intervals, effect sizes, degrees of freedom and P value noted<br>*Give P values as exact values whenever suitable.*
☒	☐	For Bayesian analysis, information on the choice of priors and Markov chain Monte Carlo settings
☐	☒	For hierarchical and complex designs, identification of the appropriate level for tests and full reporting of outcomes
☐	☒	Estimates of effect sizes (e.g. Cohen's d, Pearson's r), indicating how they were calculated

Our web collection on statistics for biologists contains articles on many of the points above.

Software and code

Policy information about availability of computer code

Data collection	No software was used
Data analysis	R Core Team (2021). R: A language and environment for statistical computing. R foundation for Statistical computing, Vienna, Austria, ULR: http://www.R-project.org.<br>Gen5, v3.05.11 (Agilent, BioTek)<br>QXONE Software, 1.4 Standard Edition (Bio-Rad)

For manuscripts utilizing custom algorithms or software that are central to the research but not yet described in published literature, software must be made available to editors and reviewers. We strongly encourage code deposition in a community repository (e.g. GitHub). See the Nature Portfolio guidelines for submitting code & software for further information.

Data

Policy information about availability of data

All manuscripts must include a data availability statement. This statement should provide the following information, where applicable:

- Accession codes, unique identifiers, or web links for publicly available datasets
- A description of any restrictions on data availability
- For clinical datasets or third party data, please ensure that the statement adheres to our policy

Provide your data availability statement here.

Research involving human participants, their data, or biological material

Policy information about studies with human participants or human data. See also policy information about sex, gender (identity/presentation), and sexual orientation and race, ethnicity and racism.

Reporting on sex and gender	We use the term sex in our study, referring to biological sex. This is necessary for data analysis.
Reporting on race, ethnicity, or other socially relevant groupings	All participants were of white-European descent. This allows for comparison with the untreated reference cohort. Data for this cohort was taken from the Latanoprost for open-angle glaucoma (UKGTS) trial - which included participants of white-European descent.
Population characteristics	A convenience sample of POAG patients was recruited from the glaucoma clinics at Moorfields Eye Hospital (London, UK) during their routine clinic appointments. Age-similar control participants were recruited from the cataract clinics at Moorfields Eye Hospital (London, UK). Ninety-nine NTG, 69 HTG and 48 Controls were recruited to Part 1 of the study. To be eligible for Part 2, participants were required to have had a minimum of 6 reliable (<15% false positive responses) visual fields over a minimum of 3 years prior to having had any form of glaucoma surgery (trabeculectomy/tube), if applicable. For eyes having had glaucoma surgery, follow-up was censored from the listing visit onward so that the observation window was immediately prior to glaucoma surgery.
Recruitment	Participants were recruited from their glaucoma clinic (POAG participants) or cataract (control participants), depending on eligibility and availability.
Ethics oversight	London- Surrey Borders Research Ethics Committee and HRA and Gwasanaeth Moeseg Ymchwil Research Ethics Service for the participant recruited in the study. Moorfields and Whittington Research Ethics Comittee for the UKEGS participants

Note that full information on the approval of the study protocol must also be provided in the manuscript.

Field-specific reporting

Please select the one below that is the best fit for your research. If you are not sure, read the appropriate sections before making your selection.

☒ Life sciences ☐ Behavioural & social sciences ☐ Ecological, evolutionary & environmental sciences

For a reference copy of the document with all sections, see nature.com/documents/nr-reporting-summary-flat.pdf

Life sciences study design

All studies must disclose on these points even when the disclosure is negative.

Sample size	The sample size calculation was based on a previous study which demonstrated lower Complex-I driven ATP synthesis in lymphocytes of NTG compared to Ocular hypertensive patients 3. The effect size was 0.47 (Cohen's (1988)). Ninety-nine patients per group were needed to detect a difference with an alpha = 0.05 and power = 0.80 (G*Power 3.1.9.2). Recruitment was impeded by the COVID-19 pandemic and concluded before our intended sample size was achieved when the ethical permissions and the designated study period expired.
Data exclusions	No participants were excluded once fulfilling eligibility requirement and consenting to participate.<br>Six high tension glaucoma and 1 normal tension glaucoma participants have no data on lymphocyte/monocyte ratio, as measured by Flow Cytometry due to experimental failure. This has been reported in the manuscript - under methods
Replication	Repeatability of the Seahorse Analyzer was done by conducting the assay on two occasions in 31 normal tension glaucoma and 12 high tension glaucoma participants.
Randomization	This was an observational, not international study, so there was no randomization.
Blinding	For the main outcome (association of basal OCR with the rate of VF loss), the author undertaking the OCR and NAD assays (explanatory variables) was masked to the visual field data (response variable). Statistical analyses were conducted by co-authors not involved in the data collection. The author undertaking the ddPCR analysis of PBMC subpopulations (response variable) was masked to diagnostic group (explanatory variable). The person undertaking the OCR and NAD assays (response variables) was not masked to diagnostic group (explanatory variable)

Reporting for specific materials, systems and methods

We require information from authors about some types of materials, experimental systems and methods used in many studies. Here, indicate whether each material, system or method listed is relevant to your study. If you are not sure if a list item applies to your research, read the appropriate section before selecting a response.

Materials & experimental systems

n/a	Involved in the study
☐	☒ Antibodies
☒	☐ Eukaryotic cell lines
☒	☐ Palaeontology and archaeology
☒	☐ Animals and other organisms
☒	☐ Clinical data
☒	☐ Dual use research of concern
☒	☐ Plants

Methods

n/a	Involved in the study
☒	☐ ChIP-seq
☐	☒ Flow cytometry
☒	☐ MRI-based neuroimaging

Antibodies

Antibodies used: CD3 Antibody, antihuman, FITC, REAfinity™ (130-113-700), REA Control Antibody (S), human IgG1, FITC, REAfinity™ (130-113-437), CD14 Antibody, antihuman, FITC, REAfinity™ (130-110-576), CD19 Antibody, antihuman, FITC, REAfinity™ (130-114-171)

Validation: Antibodies used were purchased from and fully validated by the manufacturer. Information about this, and references can be found on the manufactures website under the tabs "Extended Validation" and "References" :
For CD4: https://www.miltenyibiotec.com/GB-en/products/cd4-antibody-anti-human-reafinity-rea623.html
For CD3: https://www.miltenyibiotec.com/GB-en/products/cd3-antibody-anti-human-reafinity-rea613.html
For IgG1: https://www.miltenyibiotec.com/GB-en/products/rea-control-antibody-s-human-igg1-reafinity-rea293.html
For CD14: https://www.miltenyibiotec.com/GB-en/products/cd14-antibody-anti-human-reafinity-rea599.html
For CD19: https://www.miltenyibiotec.com/GB-en/products/cd19-antibody-anti-human-reafinity-rea675.html

Plants

Seed stocks: *Report on the source of all seed stocks or other plant material used. If applicable, state the seed stock centre and catalogue number. If plant specimens were collected from the field, describe the collection location, date and sampling procedures.*

Novel plant genotypes: *Describe the methods by which all novel plant genotypes were produced. This includes those generated by transgenic approaches, gene editing, chemical/radiation-based mutagenesis and hybridization. For transgenic lines, describe the transformation method, the number of independent lines analyzed and the generation upon which experiments were performed. For gene-edited lines, describe the editor used, the endogenous sequence targeted for editing, the targeting guide RNA sequence (if applicable) and how the editor was applied.*

Authentication: *Describe any authentication procedures for each seed stock used or novel genotype generated. Describe any experiments used to assess the effect of a mutation and, where applicable, how potential secondary effects (e.g. second site T-DNA insertions, mosiacism, off-target gene editing) were examined.*

Flow Cytometry

Plots

Confirm that:

☒ The axis labels state the marker and fluorochrome used (e.g. CD4-FITC).

☒ The axis scales are clearly visible. Include numbers along axes only for bottom left plot of group (a 'group' is an analysis of identical markers).

☒ All plots are contour plots with outliers or pseudocolor plots.

☒ A numerical value for number of cells or percentage (with statistics) is provided.

Methodology

Sample preparation: The staining process was carried out in a buffer solution comprising phosphate-buffered saline (PBS) with a pH of 7.2, 0.5% bovine serum albumin (BSA), and 2 mM ethylenediaminetetraacetic acid (EDTA), maintained on ice. After lymphoprep, cells were suspended at 1 million cells/ml in 1 ml PBS, followed by centrifugation, discarding the supernatant, and resuspending cells in 98 µl of the prepared buffer. 2ul of each antibody (separately) were then added, and the mixture was incubated in the dark at 2–8 °C for 10 minutes– antibody dilution factor 1:50. Subsequent steps involved washing (1ml of the preprepared buffer), centrifugation (300g for 10 minutes), and suspension in 1 ml PBS. Further dilution for flow cytometry was done (10 µl

of the cell suspension in 190 µl PBS), and 60 µl of the diluted suspension was loaded into a Flow cytometry cartridge for cell analysis.

Instrument

Moxi GO II™ ORFLO

Software

FlowJo™ Software v10.10

Cell population abundance

Cells were diluted as described in Sample preparations to ensure the cell number was within the linear range of the instrument,

Gating strategy

The operating principle behind the Moxi GO II Flow Cytometers is a unique combination of Coulter-style cell size determination with simultaneous fluorescence detection. As cells flow single file through the microfabricated single-use flow cell the volume of each particle is measured at the exact same time as their primary fluorescence using a 488nm solid state diode laser and 561nm/LP emission filter. Data are displayed as particle/cell size on the x-axis and fluorescence intensity on the y-axis. For the purpose of setting gates based on size the x-axis was used, while identifying where the positive population was positioned. Supplementary figure 17

☒ Tick this box to confirm that a figure exemplifying the gating strategy is provided in the Supplementary Information.
